# Large-scale assessment of 7-11-year-olds’ cognitive and sensorimotor function within the Born in Bradford longitudinal birth cohort study

**DOI:** 10.12688/wellcomeopenres.16429.1

**Published:** 2021-03-10

**Authors:** Liam JB Hill, Katy A. Shire, Richard J Allen, Kirsty Crossley, Megan L Wood, Dan Mason, Amanda H Waterman

**Affiliations:** 1 School of Psychology, University of Leeds, Leeds, West Yorkshire, LS29JT, UK; 2 Centre for Applied Education Research, Wolfson Centre for Applied Health Research, Bradford, West Yorkshire, BD96RJ, UK; 3 Born in Bradford, Bradford Institute for Health Research, Bradford, West Yorkshire, BD96RJ, UK

**Keywords:** sensorimotor, cognition, cohort, longitudinal

## Abstract

**Background: **Cognitive ability and sensorimotor function are crucial aspects of children’s development, and are associated with physical and mental health outcomes and educational attainment. The current project forms part of the Born in Bradford (BiB) longitudinal birth-cohort study, and involved measuring sensorimotor and cognitive function in over 15,000 children aged 7-10 years. This paper describes the large-scale data collection process and presents initial analyses of the data, including the relationship between cognition/sensorimotor ability and age and task difficulty, and associations between tasks.

**Method:** Data collection was completed in 86 schools between May 2016 and July 2019. Children were tested at school, individually, using a tablet computer with a digital stylus or finger touch for input. Assessments comprised a battery of three sensorimotor tasks (Tracking, Aiming, & Steering) and five cognitive tasks (three Working Memory tasks, Inhibition, and Processing Speed), which took approximately 40 minutes.

**Results: **Performance improved with increasing age and decreasing task difficulty, for each task. Performance on all three sensorimotor tasks was correlated, as was performance on the three working memory tasks. In addition, performance on a composite working memory score correlated with performance on both inhibition and processing speed. Interestingly, within age-group variation was much larger than between age-group variation.

**Conclusions:** The current project collected computerised measures of a range of cognitive and sensorimotor functions at 7-10 years of age in over 15,000 children. Performance varied as expected by age and task difficulty, and showed the predicted correlations between related tasks. Large within-age group variation highlights the need to consider the profile of individual children in studying cognitive and sensorimotor development. These data can be linked to the wider BiB dataset including measures of physical and mental health, biomarkers and genome-wide data, socio-demographic information, and routine data from local health and education services.

## Introduction

The Born in Bradford (BiB) longitudinal birth cohort study was established in 2007 to explore how behavioural, environmental, social, and genetic factors impact on developmental outcomes, including health and education ( Wright *et al.,* 2013). The study has recruited 13,776 children and their families, who reflect the city’s multi-ethnic population. BiB has produced an extensive, connected dataset including measures of physical and mental health, biomarkers and genome-wide data, socio-demographic information, as well as data linkage to routine data from local health and education services.

In 2011, as the cohort began to approach school age, measurement of children’s cognitive and sensorimotor abilities was of primary importance because these abilities are vital to educational success and to broader developmental outcomes ( Cameron *et al.,* 2012; Giles *et al.,* 2018; Lingam *et al.,* 2012; Marmot & Bell, 2012; Moffit *et al.,* 2011). Poor sensorimotor function and impairments in cognitive ability in childhood are associated with a range of adverse outcomes, such as increased mental health problems, poorer physical health (including increased risk of obesity), lower academic attainment, and poorer quality of life ( Hill *et al.,* 2016; Marteau & Hall, 2013; Stautz *et al.,* 2016; Zwicker *et al.,* 2013). Therefore, having large-scale, objective assessments of these essential skills in primary school children is invaluable to researchers, particularly given the ability to link these with the wide range of connected data available via BiB.

Consequently, between 2012 and 2014 the “Starting School” study recruited over 3,400 BiB children aged 4–5 years who were in their first year of schooling, collecting measures of school readiness including cognition and fine motor skills ( Shire *et al.,* 2020). This was followed in 2017 by the “Growing Up in Bradford” project, when the children were aged 7 to 11 years old. This large-scale multi-method programme of data collection involved children and their families in both the community and schools ( Bird *et al.,* 2019). Within this study, measuring children’s cognitive and sensorimotor function continued to be a core priority, alongside assessing social and emotional wellbeing, growth, adiposity, and cardiometabolic health.

The current paper outlines in detail the recruitment, data collection, and initial results from the school-based measures of cognition and sensorimotor skills, which were undertaken during this project. Further, because this testing was done at a whole-class level it included responses from BiB children and their classmates. Consequently, this larger dataset is also reported in this paper.

### Sensorimotor function

Sensorimotor abilities underpin our capacity to execute sensory-guided movements that achieve goal-directed actions ( Tresilian, 2012). As such, they are integral to manual manipulation, tactile and kinaesthetic processing, visual-motor integration and hand-eye coordination ( Goyen *et al.,* 2011; Krakauer & Mazzoni, 2011; Snapp-Childs *et al.,* 2013). Proficient sensorimotor control is essential for a plethora of everyday tasks such as getting dressed, using cutlery, and manipulating a pen or pencil in the classroom ( Miller *et al.,* 2001; Prunty *et al.,* 2013; Wang *et al.,* 2009). These abilities develop throughout childhood ( Flatters *et al.,* 2014; Sugden *et al.*, 2013), with theory suggesting that competent sensorimotor control is vital for one’s ability to interact with, and understand the environment through “purposeful, coordinated movements” ( Latash, 2012, p. 1; Piaget, 1952). For example, basic sensorimotor abilities in infancy and early childhood are associated with emerging cognitive abilities, such as sustained and joint visual attention and inhibitory control ( D’Souza *et al.,* 2017; Gottwald *et al.,* 2016; Yu & Smith, 2017; Yuan *et al.,* 2019). There is also evidence for the importance of sensorimotor function later in childhood with studies showing the importance of the role of action in learning processes and the retention of information ( Gathercole *et al.,* 2008; James, 2017; LeBarton *et al.,* 2015; Waterman *et al.,* 2017; Yang *et al.,* 2017).

Meanwhile, difficulties with sensorimotor control are documented as limiting the development of perception, cognition and motivation ( Leonard, 2016; von Hofsten, 2004). Coordination difficulties are also associated with an abundance of adverse developmental outcomes, including lower levels of physical activity ( Kwan *et al.,* 2016), obesity ( Augustijn *et al.,* 2018), social and emotional wellbeing ( Hill *et al.,* 2016), quality of life ( Zwicker *et al.,* 2013), and academic attainment ( Harrowell *et al.,* 2017).

Moreover, whilst clinically significant problems with sensorimotor control are associated with a concurrent increased risks of mental health problems ( Lingam *et al.,* 2012), difficulties with sensorimotor control are also frequently found to co-occur in children already diagnosed with a range of other specific genetic ( Cunningham *et al.,* 2019; Cunningham *et al.,* 2020) and developmental disorders ( Pieters *et al.,* 2012).

The current project assesses sensorimotor function by recording end-point kinematic data on participants’ movements as they performed three separate tasks that are representative of fundamental coordinative abilities: tracking, aiming, and steering. Tracking relies on the ability to predict target movement and perform a sustained, rhythmic visuo-manual tracking behaviour. Aiming movements rely on accurate feed forward mechanisms and fast implementation of online corrections to produce simple ballistic responses (e.g. reach to grasp behaviours). Lastly, steering requires precise force control to plan and produce multi-component movements, integrating these components proficiently under timing constraints.

### Cognitive function

Cognitive skills are equally essential for academic success ( Ahmed *et al.,* 2019; Alloway *et al.,* 2009; Blakey *et al.,* 2020; Blair & Razza, 2007; Lee *et al.,* 2011) and are linked to outcomes such as social functioning ( McQuade *et al.,* 2013) and long term health ( Marteau & Hall, 2013; Stautz *et al.,* 2016). In the current study we assessed three core components of cognition: working memory, inhibitory control, and processing speed. The rationale for focussing on each of these areas is discussed in the following sections.

***Working memory.*** Working memory is a limited capacity system that is used to store and process information for immediate use in ongoing cognitive activity ( Baddeley *et al.*, 1986; Cowan, 1999). Working memory ability increases throughout childhood and adolescence, reaching adult-like levels at approximately 15 years of age ( Gathercole *et al.,* 2004). Working memory is essential for learning and predicts educational achievement including attainment in reading, mathematics and science ( Alloway *et al.,* 2014; Cragg *et al.,* 2017; Gathercole *et al.,* 2004; Holmes & Adams, 2006; Monette *et al.,* 2011; Swanson *et al.,* 2006). Impairments to working memory also co-occur with several developmental disorders, for example, attention deficit hyperactivity disorder (ADHD), developmental coordination disorder (DCD), and dyslexia ( Alloway & Archibald, 2008; Beneventi *et al.,* 2010; Martinussen *et al.,* 2005; Smith-Spark & Fisk, 2007). We measured working memory using three separate tasks: forward digit recall (FDR), the Corsi task, and backward digit recall (BDR). FDR measures the ability to retain verbal information, Corsi measures the ability to retain visuospatial information, and BDR taps into the ability to engage in executive control of information. Each of these tasks is used extensively in the working memory literature ( Alloway *et al.,* 2006; Berry *et al.,* 2018; Berry *et al.,* 2019; Gathercole *et al.,* 2004; Waterman *et al.,* 2017).

***Inhibitory control.*** Inhibitory control refers to the ability to suppress prepotent responses and ignore irrelevant or distracting information. It is one of the central constructs of executive function ( Diamond, 2013). Indeed, some researchers have argued that working memory and inhibition are the two key factors underlying executive function ( Roberts & Pennington, 1996; Senn *et al.,* 2004). The ability to inhibit irrelevant information and actions increases over childhood ( Diamond & Taylor, 1996; Durston *et al.,* 2002; Williams *et al.,* 1999). Improved inhibitory control is linked to academic attainment ( Blair & Razza, 2007; Kieffer *et al.,* 2013; Szucs *et al.,* 2013) and to broader long-term health outcomes such as body mass index (BMI) and alcohol use ( Marteau & Hall, 2013; Moffit *et al.,* 2011; Stautz *et al.,* 2016). The inhibition task we used was based on a classic Flanker design ( Blakey & Carroll, 2015; Eriksen & Schulz, 1979; Rueda *et al.,* 2005).

***Processing speed.*** Processing speed is a fundamental part of cognition ( Kail & Salthouse, 1994), enabling increased efficiency and improved performance on other cognitive tasks. Speed of processing increases considerably during early and middle childhood, with rate of increase slowing during late childhood, and then stabilising by late adolescence ( Kail, 1991; Kail & Ferrer, 2007). Improved processing speed is linked to broader academic attainment ( Gordon *et al.,* 2018; Mulder *et al.,* 2010; Rohde & Thompson, 2007), as well as to reading ( Kail & Hall, 1994; Lobier *et al.,* 2013) and mathematics ( Geary, 2011; Geary & Brown, 1991). Poor processing speed also co-occurs with many learning difficulties such as ADHD and Autism ( Dickerson Mayes & Calhoun, 2007). The measure used in the current study was based on a speeded counting task methodology that has been used previously ( Fry & Hale, 1996; Weiler *et al.,* 2003).

### General aims and hypotheses

The current project aimed to collect data on sensorimotor and cognitive function, using objective computerised measures, on approximately 6,000 BiB children attending Bradford Primary Schools (covering ages 7–11 years) as well as their classmates (N ~ 9,000). Cross-sectional data on this scale on children’s sensorimotor and cognitive functions is, in and of itself, valuable to researchers interested in exploring the relationships between these domains. Moreover, for BiB children involved in this study their data can also be linked to the further genetic, environmental, demographic, socioemotional, health, and educational data collected as part of the broader cohort study. Thus, providing opportunities to improve our understanding of the complex interplay of factors affecting child development.

The current paper describes the data collection process for the school-based assessment of sensorimotor and cognitive function in detail and presents an initial analysis of the data. The key research questions studied in relation to each task are:

1. How does performance change with age?
2. How does performance change with increasing difficulty within each task?
3. What are the associations between performance across the different tasks, within each of the two key domains (I.e. cognition and sensorimotor ability)?

## Methods

### Setting

***Dates.*** Data collection for the study took place across four academic years (May – July 2016 and September – July 2016/17, 2017/18, and 2018/2019).

***Location.*** Over the period of data collection, the Bradford District (West Yorkshire) was the fifth-largest metropolitan district in the UK (over half a million people), with nearly a quarter (23.7%) of residents aged under 16 years old ( Office for National Statistics, 2019). Over the years, between 22 and 29% of the district's children were classified as living in Poverty ( Office for National Statistics, 2019; The English Indices of Deprivation, 2019). During this time, around 64% of the Bradford District’s population self-identified as being of White British ethnic origin, with a further 20% identifying instead as Pakistani.

### Participants

***Schools.*** *Eligibility:* Schools in the Bradford district were initially invited to participate based on whether they had participated in a previous study called ‘Starting School’ that was carried out when the BiB children were in Reception year of school (ages 4-5) ( Shire *et al.,* 2020). Additional schools were invited based on the team’s capacity, starting with those schools that had the highest numbers of children attending who were already part of the BiB study.

*Method of recruitment:* BiB researchers sent out individual emails addressed to Head teachers (or a key member of staff identified during previous years’ recruitment), with an information sheet attached. If no response was received, telephone calls were made and, if desired and feasible, a member of the research team would organise to meet a member of staff to explain the project. Recruitment of schools recurred annually over the 4 years of the study and if schools did not respond one academic year they were still contacted again in subsequent years, unless they requested otherwise. Head teachers had to provide written, opt-in consent on behalf of their school in order to participate.

***Children.*** *Eligibility:* the intention was to assess children as close as possible to when they were 8 years old. There were three stages to recruitment. During a pilot phase between May 2016 and July 2016, only children in Year 4 (ages 8–9 years old) were invited to take part. In the main phase of recruitment, between September 2016 and July 2018, children in Years 3 (ages 7–8 years old) and 4 were eligible. If we had visited a school in the previous year, we would only capture data from the new Year 3 children the next academic year. In the final phase of recruitment (September 2018 – July 2019) children in Years 3, 4 and 5 were eligible in newly recruited schools for that year.

*Method of recruitment:* Recruited schools were given information sheets and opt-out consent forms to distribute to parents of children in the eligible year groups, which went out one to two weeks ahead of the school visit. The opt-out consent approach had been successfully used in the previous BiB Starting School study ( Shire *et al.,* 2020), where it was chosen due to the low-risk nature of participation, the high numbers of children targeted, and the risks of excluding groups of children from homes where school forms are frequently not returned. One school asked for opt-in consent forms to be used and these were provided.

Parents were asked to return the opt-out consent forms to the school, and these were collected by researchers at the start of a visit. Only a small minority of parents withdrew their child from the study. For example, in the final phase of recruitment (Sept 18 – July 19), out of 5570 parents of children approached, 252 opted-out of the study (4%). Two further pathways by which a child could be withdrawn from the study also existed. Firstly, on a few occasions, parents would verbally inform their child’s teacher that they did not wish their child to take part. In these circumstances the teacher’s verbal account of opt-out for that child was recorded, and the child was not assessed. Secondly, Teachers would also occasionally inform the researchers that they felt that a child’s special education needs (SEN) would prevent them from participating; we took the teachers decision on whether the child should participate or not. Assent from the children was also obtained before every assessment to ensure the child was happy to take part. Again, refusal at this stage was also rare. In the final phase of recruitment mentioned previously, only six children refused to take part in the assessments.

### Design and general procedure

***Ethical approval.*** Ethical approval for this study was obtained from the NHS Health Research Authority’s Yorkshire and the Humber - Bradford Leeds Research Ethics Committee (reference: 16/YH/0062) on the 24 ^th^ March 2016.

***Design and measurements.*** For both the sensorimotor and cognitive measures, data collection for this cross-sectional study took place during school time. All the assessments were presented on a tablet computer (Lenovo ThinkPad Helix type 20CG or 20CH) that used a digital stylus and finger touch for input. The children were presented with the sensorimotor tasks first (Tracking, Aiming and Steering) followed by the cognitive tasks (Forwards Digit Recall, Backwards Digit Recall, Corsi Block test, Flanker Task, and Processing Speed). The procedure was very similar to the one used for the same tablet-based sensorimotor assessments during the Starting Schools study (methods described in Shire *et al.,* 2020). The only difference being an additional 20–25 minutes of cognitive tasks on the same tablet computers. The children interacted with the visual stimuli on the touch screen tablet using a digital stylus for the sensorimotor tasks and finger touch for the cognitive tasks. Tasks were presented in the same order to all children, following standardised instructions and a practice trial.

We offered schools the opportunity for both BiB children and their classmates (non-BiB children) to take part in the study. It was up to the school's discretion whether they preferred just BiB children to take part, to minimise the impact on the school day. In the majority of cases, schools opted for both BiB children and non-BiB children to be tested.

***Procedure.*** Once the school consented to participate, they were asked to provide class lists ahead of the school visit that included: children’s name; date of birth; Unique Pupil Number (UPN) ^1^; home post code; gender and ethnicity. This information was used to allow us to identify children eligible for assessment within their classes and to link the data collected back to the wider BiB cohort database, for those children who were part of the cohort. It also provided a means to resolving data entry errors (e.g. if the UPN was missing then linkage could be made using other identifiable information, reducing loss of data). Data was collected and stored securely following the Bradford Teaching Hospitals Foundation Trust procedures, which helps to protect all the BiB studies and is in accordance with the Data Protection Act 1998. Transfer of data onto the secure central archive at the BiB research office was via encrypted devices (e.g. tablets, memory sticks) or encrypted emails.

Schools were asked to allocate a quiet room or area within their premises for the research team to use to conduct their assessments (e.g. an empty classroom). Typically, a team of five researchers took 10 children out of class at a time, with a ratio of no more than two children to one assessor. The assessor would administer the sensorimotor and cognitive assessments to the children, explaining what was required of each task at the beginning of each assessment and ensuring the child understood. Assessments took approximately 40–45 minutes per child.

Senior staff within the BiB cohort study trained the assessment team. Refresher training was provided at the start of each academic year and when required throughout the year to ensure high compliance with procedural protocols.

***Feedback to schools.*** The results of the sensorimotor and cognitive assessments were compiled into individual reports for each child, and delivered back to class teachers, along with a document explaining why these skills were developmentally important, how the children were assessed, what was measured, what the scores meant and how they should be interpreted. These feedback documents were used at the discretion of the school, typically in conjunction with their own data on each child, to consider possible further assessment and evaluation for some children.

### Sensorimotor methods

**Figure 1.**
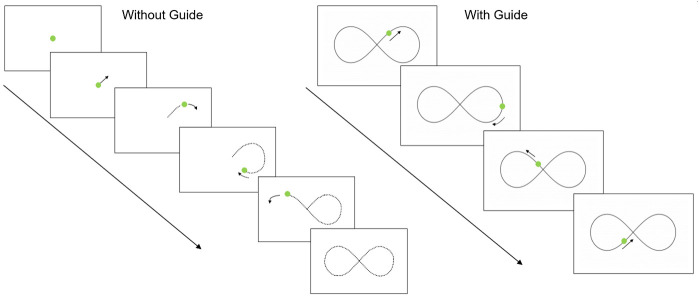
Sensorimotor battery: Tracking task. ‘Without Guide’ sequence demonstrates the without-guide tracking trial (the dotted line indicates the trajectory the target is moving but these prompts are not visible to participants). “With Guide” sequence shows the tracking trial with the visible guide added.

***Materials and procedure.*** The Clinical-Kinematic Assessment Tool (CKAT), an objective computerised assessment, was used to record sensorimotor function (see Culmer *et al.*, 2009 for full technical details). This battery contains three tasks (tracking, aiming and steering), and takes approximately 12–15 minutes in total to administer. In all tasks, the children used a pen-shaped stylus to interact with the touchscreen tablet and movement of the stylus is recorded at 120 Hz, allowing task-relevant kinematic features of these movements to then be calculated. For example, temporal indices can measure the elapsed time between presentation of on-screen stimuli and consequent actions being initiated (Reaction Time; RT); and then the time between initiation and the end of the subsequent action (movement time; MT). Combining RT and MT then gives a measure of Total Response Time (TRT). Efficiency and accuracy of movements are described using spatial indices. These includes Path Accuracy, which compares the recorded trajectory produced by participant to a model trajectory. Finally, dynamic indices summarise speed/accuracy trade-offs in sensorimotor control. For example, root-mean-square error (RMSE) measures average spatial error whilst tracking a target over time, and penalised Path Accuracy (pPA) records path accuracy after adjusting for length of movement time. Consistent with procedures in Flatters *et al.* (2014), one kinematic metric per CKAT task was selected, to be used as an index of task performance (RMSE for Tracking, Total Response Time for Aiming and Penalised Path Accuracy for Steering). A brief description of each task follows here, along with illustration of these tasks in Figure 1– Figure 3. For a fuller description see Flatters *et al.,* 2014:

**Figure 2.**
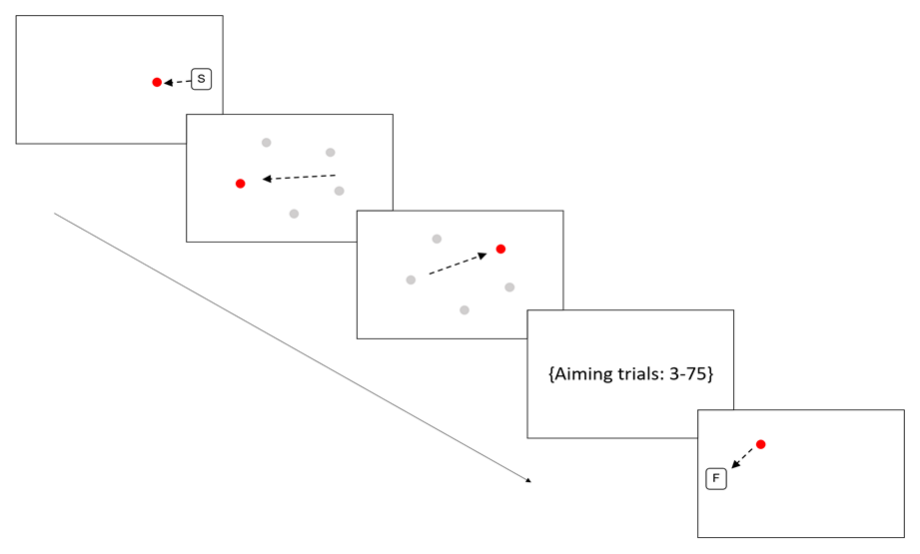
Sensorimotor battery: Aiming task. Arrows are indicative of the direction of movement the participant should make; these arrows would not be visible to the participants. The position of the five targets is shown in grayscale but again these would not be visible to participants. Figure displays first two aiming trials only.

**Figure 3.**
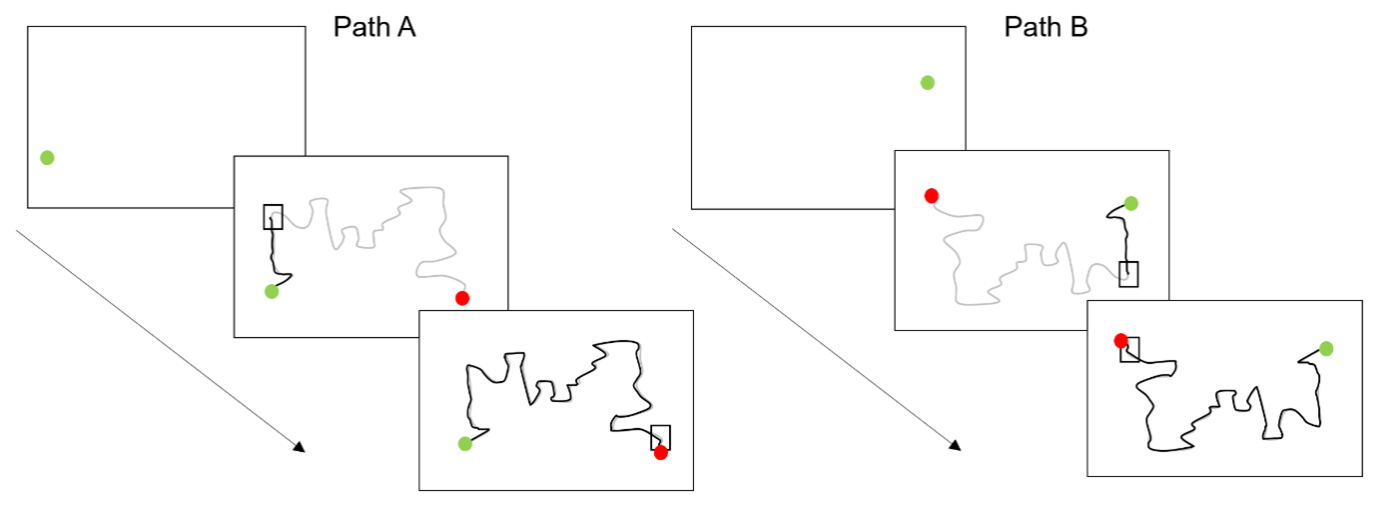
Sensorimotor battery: Steering task. Shows path **A** and path **B** sequences. The thick grey line shows the path participants were expected to trace. The thick black lines demonstrate the actual tracing path participants made. The square box represents the “pacing box” which participants were expected to stay within.

Tracking

A moving green circle is presented on the screen and participants are required to track the circle around the screen, keeping the tip of the stylus within the circle (which moved in a ‘figure-of-eight’ pattern for nine revolutions). The speed of the moving circle increases every three revolutions, producing Slow, Medium, and Fast conditions (42, 84, and 168 mm/s, respectively). This task comprises of two consecutive conditions; an unguided condition (Without Guide) where the path could not be seen and a guided trial (With Guide; where the ‘figure-of-eight’ is displayed on the screen). Each trial lasts approximately 84 s. See Figure 1 for illustration.

Aiming

A series of green circles are presented on the screen and the child is required to move the stylus as quickly as possible (without disconnecting the stylus from the screen) from one green circle to the next, as they appear. Each time the tip of the stylus reaches a green circle, that circle disappears, and a new target circle appears in a new location. A total of 75 discrete aiming movements are made taking approximately 2–4 minutes. In six of the final 25 movements the target shifted position (referred to Jump trials) after the participant initiated movement and got within 4 cm of the initially aimed at target, forcing the participant to make an online correction to ‘jump’ to the new target location. All targets appeared at a fixed distance from each other. See Figure 2 for illustration.

Steering

The steering task comprised of two trials, Path A and Path B (Path B was the reverse order of path A) and each took approximately 40 s to complete. This task was shortened to two trials instead of six, compared to the version used in Flatters *et al.,* 2014, due to time constraints. A 5-mm-wide pathway consisting of two parallel lines was presented on the screen, running from a ‘start’ to a ‘finish’ position on the other side of the screen. Children are instructed to keep the tip of the stylus inside the pathway whilst also keeping their stylus within a ‘pacing box’ (sequentially moved along the path in 5-s intervals). This pacing box attempts to standardise the pace of movement and encourage a greater focus on accuracy during this set time period. See Figure 3 for illustration.

***Domain-specific hypotheses.*** Consistent with response patterns reported previously in a much smaller sample of 7–11-year-olds (see Flatters *et al.,* 2014), it was predicted that with increasing age, performance across all tasks would significantly improve. For Tracking specifically, improved RMSE was also predicted within the guided condition and at slower target speeds. These effects were also expected to interact with each other, whereby the positive effect of including the visual guide would be larger at slower speeds and this advantage would be more evident in older age groups. Within the Aiming task specifically, slower total response time was expected within Jump trials compared to ‘baseline’ trials, where no online correction was required. However, no interaction of this effect with age was predicted. Lastly, in the Steering task only an effect of age was predicted, with no difference in Penalised Path Accuracy as a function of condition expected (Path A versus Path B). Lastly, it was expected that there would be a significant correlation between performance on each of the three sensorimotor tasks.

### Cognitive methods

***Materials and procedure.*** Working memory, inhibition and processing speed were assessed through five cognitive tasks, which took approximately 20–25 minutes to complete. Working memory was assessed using three different tasks: Forward Digit Recall (FDR), Backwards Digit Recall (BDR) and the Corsi task (Corsi). Inhibition was assessed using a Flanker task. The order of presentation of these tasks were as follows: FDR, BDR **,** Corsi, Flanker task, and Processing Speed (PS).

FDR

**Figure 4.**
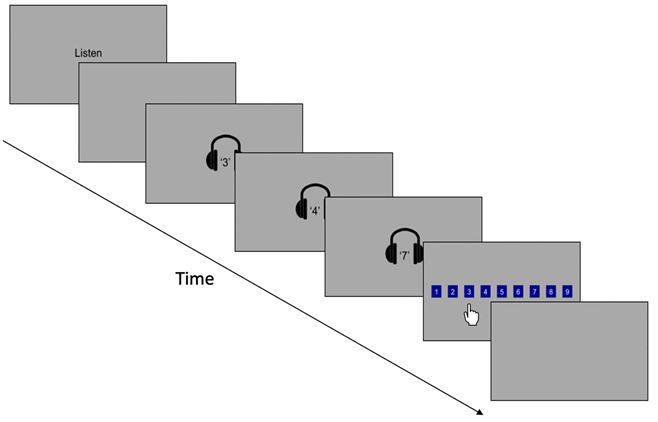
Schematic illustration of the forwards digit recall working memory task.

Children were presented with a sequence of numbers through headphones and subsequently asked to recall these numbers in the order they were audibly presented, by touching the appropriate boxes on the screen in order (see Figure 4). Nine boxes were ordered sequentially from 1 to 9 on the screen. The tasks progressed from sequence length three to six, with four trials for each sequence length, with a total of 16 trials. Response accuracy (correct or incorrect) and reaction time (s) was recorded for each trial. For each sequence length, the primary outcome variable was mean proportion correct and the secondary outcome variable was mean reaction time (RT).

BDR

This task was similar to FDR but this time children were asked to recall the numbers in reverse order. As this task is more difficult than FDR, sequence length started at two digits and increased to sequence length five, with four trials at each length. The same outcome variables were recorded for this task as for FDR.

Corsi block tapping

**Figure 5.**
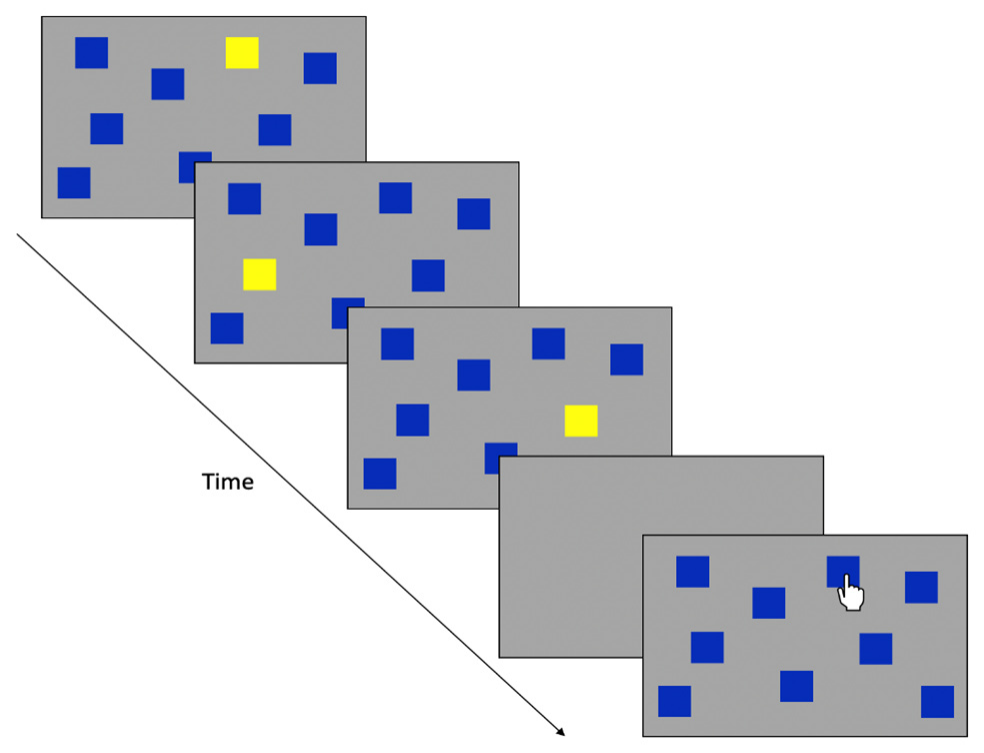
Schematic illustration of the Corsi spatial working memory task.

Children were presented with nine randomly arranged blue squares, in which a random and unique sequence of boxes flashed yellow. The task was for the child to remember the order and once the sequence was finished, to tap the blue boxes in the order in which the yellow boxes flashed (see Figure 5). Sequence length increased as the task progressed, from three squares to six squares, with four standardised sequences presented for each sequence length, equalling a total of 16 trials. Both response accuracy (correct and incorrect) and reaction time (s) were recorded for each item. Mean proportion correct was the primary outcome and mean RT was the secondary outcome variable for each sequence length.

Inhibition (Flanker task)

**Figure 6.**
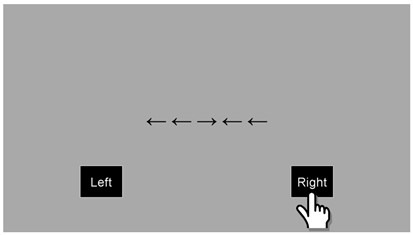
Schematic illustration of the inhibition (Flanker) task, showing an incongruent trial.

A line of five arrows was presented to the children in the centre of the screen. Children were required to identify the direction of the middle arrow, whilst the surrounding four arrows were either pointing in the same (congruous) or an opposite (incongruous) direction to the middle arrow (see Figure 6). There was a total of four practice trials and 40 test trials. In each case, these were equally balanced between congruous and incongruous trials, with an equal number to the left and right. The children were asked to answer as quickly and accurately as possible. Accuracy and RT were captured, with the primary outcome variable being: Mean (RT to congruous trials – RT to incongruous trials).

Processing speed

**Figure 7.**
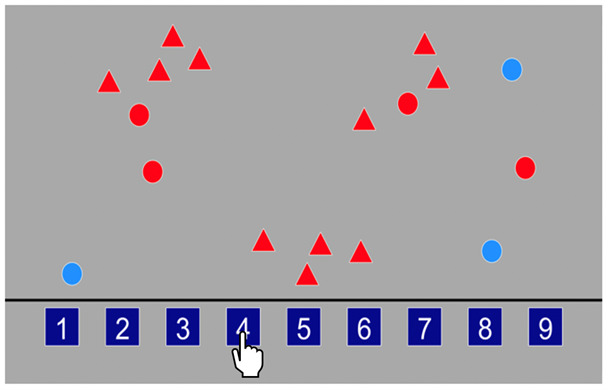
Schematic illustration of the processing speed task.

Children were asked to identify how many red circles were present on the screen, amongst a random number of red triangles and blue circles, and to respond by tapping the box located at the bottom of the screen containing the correct number (see Figure 7). The boxes at the bottom of the screen included number options 1-9. There was a total of 18 trials and the children were asked to carry out each trial at quickly and as accurately as possible. Response accuracy (correct and incorrect) was recorded for each trial as well as RT (s). The primary outcome variable was mean RT for correct trials.

***Domain-specific hypotheses.*** For the three working memory tasks, accuracy was expected to improve with age (e.g. Gathercole, 1999; Gathercole *et al.,* 2004). Due to the increased processing demands involved in reversing the digit sequence for recall (e.g. Alloway *et al.,* 2006), BDR was expected to be more difficult than FDR or Corsi and result in lower accuracy overall ( Anders & Lillyquist, 1971). For each of these three tasks, performance was predicted to decline as sequence length increased, in line with the view of working memory as a limited capacity system (e.g. Cowan, 2001).

For the inhibition and processing speed tasks, children were expected to show faster reaction times with increasing age. Accuracy was a secondary dependent variable in these tasks and was expected to be high, but with similar age-related improvements apparent. For the inhibition task, responses were predicted to be faster (and more accurate) for congruent trials, relative to incongruent trials ( Blakey & Carroll, 2015; Eriksen & Eriksen, 1974; Mullane *et al.,* 2009). This was expected to be more apparent for the younger age groups, indicating developmental changes in executive control and inhibition with age ( Diamond, 2013).

Finally, the interrelationships between the different tasks at an individual differences level was explored. We expected positive correlations between each of the working memory measures, particularly for FDR and BDR, in line with the concept of shared processing components alongside domain-specific storage ( Alloway *et al.,* 2006). We also predicted positive correlations between a working memory composite score, reaction time on the inhibition and processing speed tasks, and the magnitude of the congruency advantage within the inhibition task.

### Data analysis

***Data cleaning and preparation.*** Prior to analyses, the sensorimotor and cognitive data collected was reviewed for quality control purposes. Sessions of recorded data were omitted for one of three primary reasons: duplication; incompleteness or issues raised in an accompanying field-note ^2^.

Duplicated sessions were cases in which the identifying information for the participant was the same across multiple sessions, this sometimes also included the recorded cognitive and/or sensorimotor data also being identical. In cases of complete duplications (i.e. identifying information and recorded data were identical), one session was retained, and the other(s) omitted. In cases where only the identifying information but not the data were identical this likely occurred as a result of human error in data entry and so both/all occurrences were omitted. An exception to this was where a child began but did not complete data collection within one session but continued it in a second session. In these cases, if there was also a field note confirming this was the case, then these two sessions were combined.

Incomplete cases arose where data was missing for some tasks or some trials within tasks. Participants had to have complete data for a task for it to be included in analysis. For example, if trials were incomplete or missing entirely for a participant’s Tracking task but were complete for their Aiming, Steering, and Cognitive Tasks then only their Tracking data would be omitted. For this reason, the sample sizes for each task varies somewhat, presented in Table 1.

**Table 1.** Number of participants with data for each of the Sensorimotor and Cognitive tasks, respectively stratified by age group.

	Total sample	7 Years	8 Years	9 Years	10 Years
**Sensorimotor**					
Tracking	15701	5217	6985	3203	296
Aiming	15727	5230	6995	3206	296
Steering	15720	5217	6998	3208	297
**Cognitive**					
FDR	15473	5154	6870	3152	297
BDR	15146	5000	6725	3126	295
Corsi	15366	5095	6826	3146	299
Inhibition	14051	4634	6199	2927	291
Processing Speed	14933	4915	6633	3086	299

FDR=forward digit recall; BDR=backwards digit recall

Lastly, field notes were inspected and judged on a case by case basis by two researchers, with data from these sessions excluded if either researcher they felt the nature of the note warranted exclusion. Examples of notes which led to exclusion include technical difficulties (i.e. tablet crashes) or indications that participants were non-compliant with instructions.

Each dataset was analysed using mixed methods ANOVAs with Bonferroni-corrected follow-up comparisons. This was then supplemented by correlational analyses between separate sub-tests within both of the two key domains (sensorimotor and cognitive).

## Results

### School recruitment

**Figure 8.**
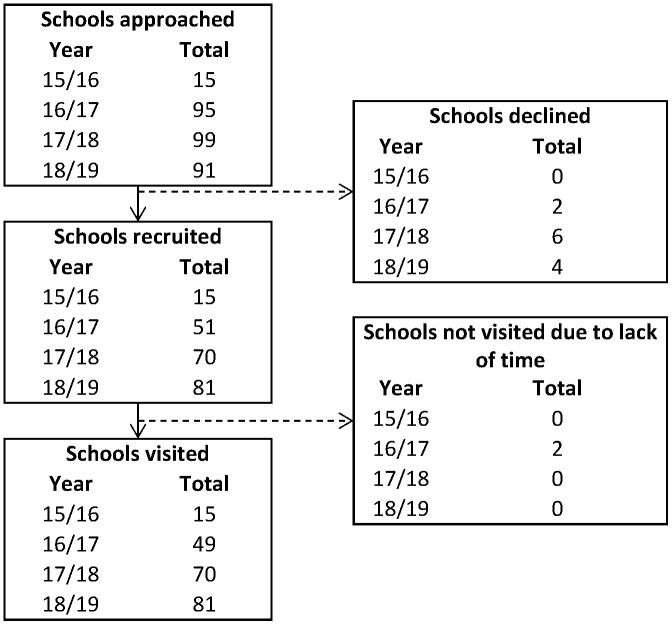
Flowchart illustrating school participation from recruitment through to being visited by the research team.

Over the four testing periods, 86 schools in Bradford participated (see Figure 8 for breakdown of number of schools per year). In the school year 2016/2017, two schools consented and visits arranged but these were subsequently cancelled by the schools (due to other demands). These were unable to be re-booked that year due to capacity of the research team, but all three schools were all visited the following year. A total of 12 schools took part in all four testing periods, 35 in three of the periods, 23 in two, and 16 in only one testing period.

### Child recruitment

Over all three phases of testing, 17,774 children were recruited to take part (i.e. were in an eligible year group in a consenting school, and whose parents had not opted them out of the study), see Table 2 for a breakdown of children consented each year.

**Table 2.** Numbers of children recruited and subsequently assessed, by year.

School year	Sample	Children recruited	Children assessed	% of total assessed
2015–2016	BiB	832	717	86.18
	Non-BiB	110	102	92.73
	Total	942	819	86.94
2016–2017	BiB	3189	2821	88.46
	Non-BiB	2773	2490	89.79
	Total	5962	5311	89.08
2017–2018	BiB	3015	2671	88.59
	Non-BiB	2537	2285	90.07
	Total	5552	4956	89.27
2018–2019	BiB	3856	3395	88.04
	Non-BiB	1462	1339	91.59
	Total	5318	4734	89.02
**Total**	**BiB**	**10892**	**9604**	**88.17**
	**Non-BiB**	**6882**	**6216**	**90.32**
	**Total**	**17774**	**15820**	**89.01**

BiB=Born in Bradford cohort

Not all of these children completed the testing, however, due to either being absent on the day, refusing to take part, or through there being not enough time during the visit to complete testing with all the children. A breakdown of number of children falling into each of these groups was only captured during the final phase of testing (school year 2018–2019). In this year, 242 children were absent (62 BiB), 6 children refused to take part (3 BiB), and 69 (12 BiB) were not tested due to running out of time during the school visit.

### Sensorimotor measures

See the data availability statement for the link to the script used to derive the three variables used within this paper (RMSE, TRT and pPA) from the raw output produced by CKAT. Additional processing of these variables was then conducted (negative reciprocal transformation) to normalise the distribution.

**Figure 9.**
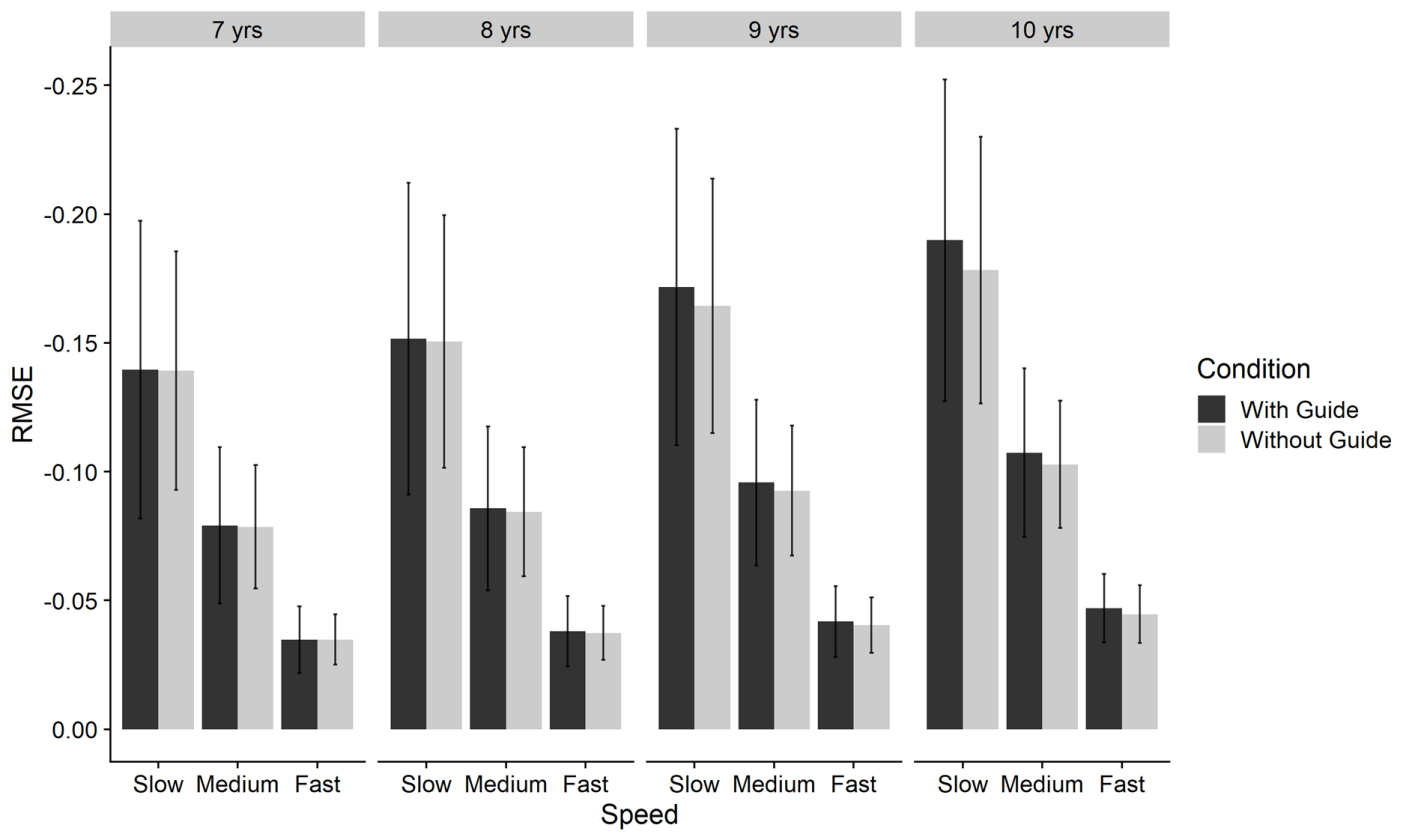
Bar chart of negative-reciprocal Root Mean Squared Error (RMSE) by age-group, condition and speed for Tracking task. Error bars denote SD. Note: Higher score = increased accuracy.

***Tracking.*** A 2x3x4 mixed ANOVA was conducted with condition (With Guide versus Without Guide) and speed (Slow, Medium, and Fast) as within-groups factors and age-group (7-, 8-, 9- and 10-year olds) as the between-subjects factor. Significant main effects (all p<.001) of age-group ( $\eta_{p}^{2}$ = .063), condition ( $\eta_{p}^{2}$ = .004), and speed ( $\eta_{p}^{2}$ = .642) on negative reciprocal RMSE were noted. As was a significant three-way interaction ( $\eta_{p}^{2}$ = .001), illustrated in Figure 9.

Post-hoc comparisons (Bonferroni-corrected) indicated significant differences between all age groups, with greater performance for 10-year-olds (M = .112, SE = .001), compared to 9-year-olds (M = .101, SE < .001), 8-year-olds (M = .091, SE < .001), and 7-year-olds (M = -.084, SE < .001). Speed related effects were attenuated in younger age groups and for 7-year-olds and in younger age groups there was little effect of condition in any of the three speeds. However, with increasing age, there were larger effects of condition, supporting the significant 3-way interaction that was found.

***Aiming.*** A 2x4 mixed ANOVA was carried out with condition as the within-groups factor (Baseline versus Jump) and age group as the between-subjects factor. This revealed significant main effects (at p < .001) of age group ( $\eta_{p}^{2}$ =.078) and condition ( $\eta_{p}^{2}$ =.910) and the age group x condition interaction ( $\eta_{p}^{2}$ =.048).

**Figure 10.**
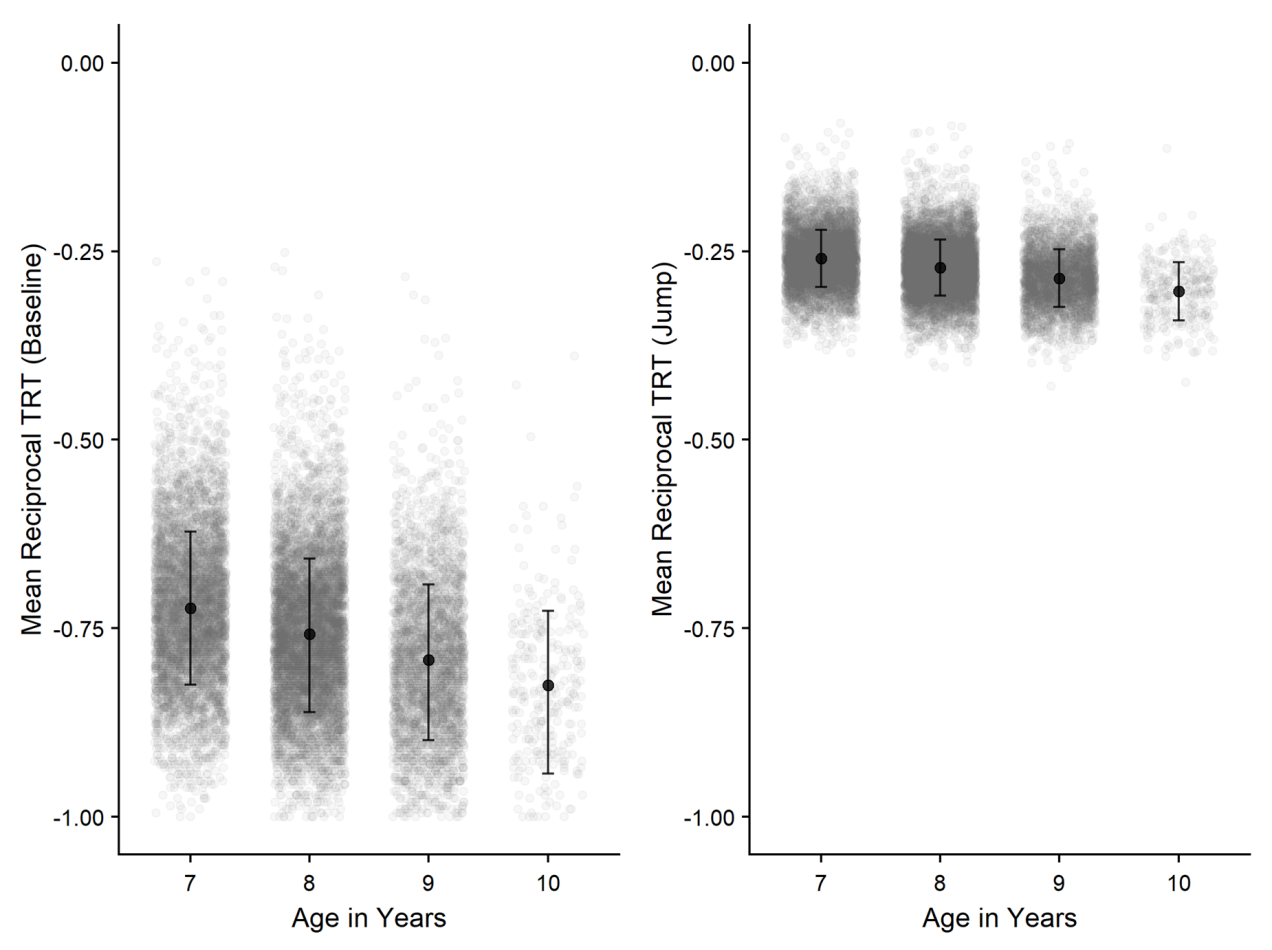
Negative-reciprocal total response time (TRT) by age-group and condition for Aiming task. Error bars denote SD and grey points denote individual children. Note: Lower score = faster responses.

Further comparisons (Bonferroni-corrected) revealed that performance between all age groups significantly differed from each other with 10-year-old children performing the best. Age group effects observed were larger within the Baseline condition compared to the Jump condition, explaining the significant age-group x condition interaction found, illustrated in Figure 10. In addition, greater differences were found between the Baseline and Jump conditions with increasing age.

***Steering.*** A similar 2x4 mixed ANOVA was conducted for the Steering task with condition as the within-groups factor (Path A versus Path B) and age group as the between-subjects factor. Significant main effects (at p <.001) were found for age group ( $\eta_{p}^{2}$ =.032) and condition ( $\eta_{p}^{2}$ =.003) However, a significant interaction was not found (p = .269).

**Figure 11.**
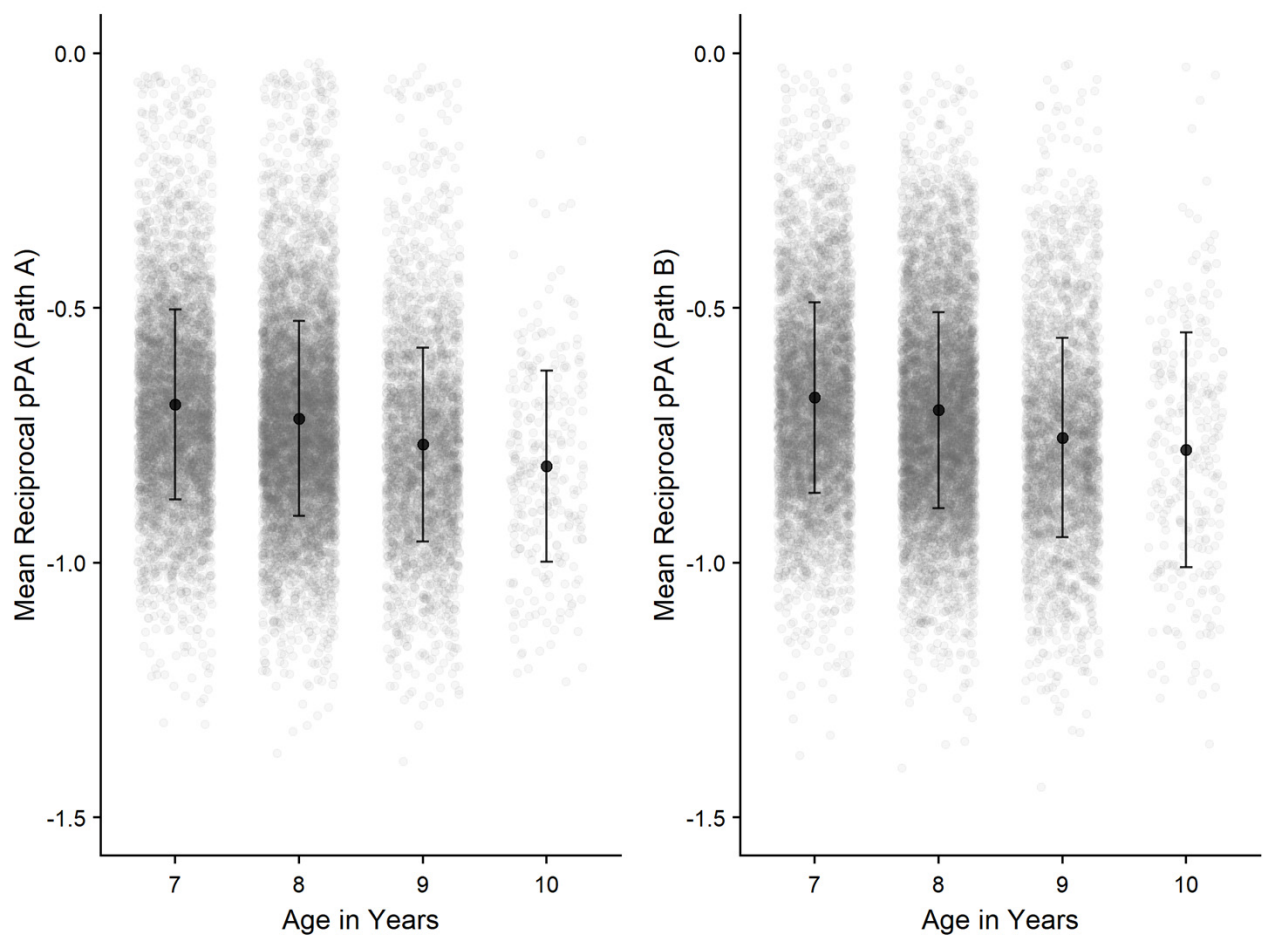
Negative-reciprocal penalised path accuracy (pPA) by age-group and condition for Steering task. Error bars denote SD and grey points denote individual children. Note: Lower score = increased accuracy.

Further comparisons (Bonferroni-corrected) showed performance across groups significantly differed from each other (all *p* <.001 except between 9-year-olds and 10-year-olds, *p* <.01). Whilst children were found to perform significantly better on Path A compared to Path B ( *p* <.001) ( Figure 11), the effect size was comparatively small, indicating minimal impact in comparison to the effect of age on performance.

**Figure 12.**
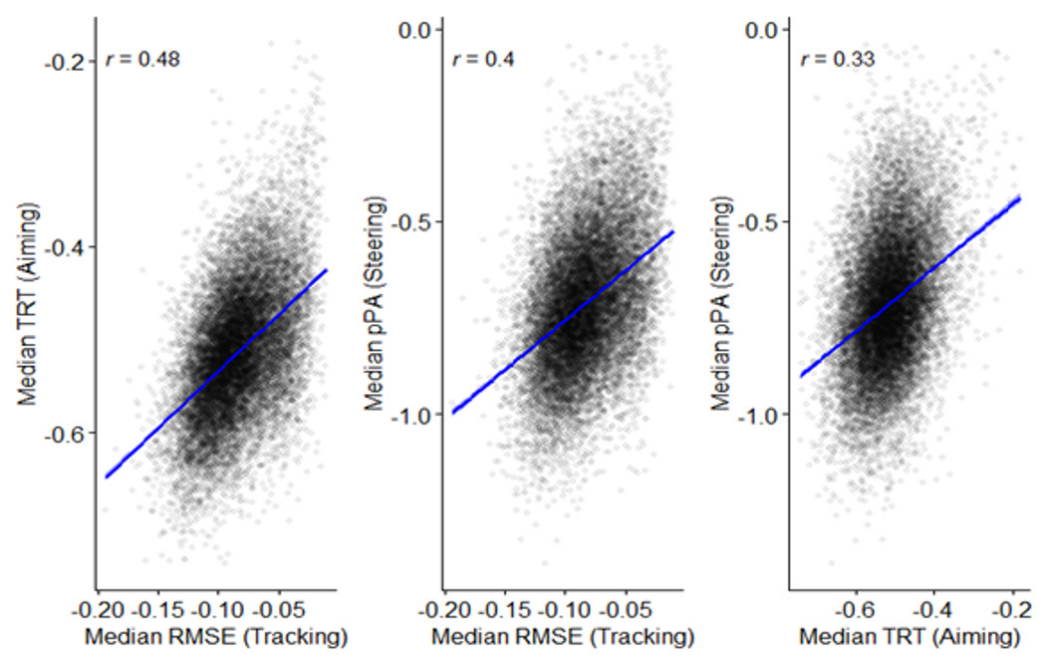
Scatterplots and correlations (Pearson’s correlation coefficients) illustrating the relationship between the three sensorimotor tasks (N=15,817).

***Relationships across sensorimotor tasks.*** Correlational analyses were conducted to observe how performance is related across the three CKAT tasks. Figure 12 show the interrelationships between Tracking, Aiming, and Steering. Positive correlations (significant at p <.001) are apparent between all three sensorimotor tasks.

### Cognitive measures

**Figure 13.**
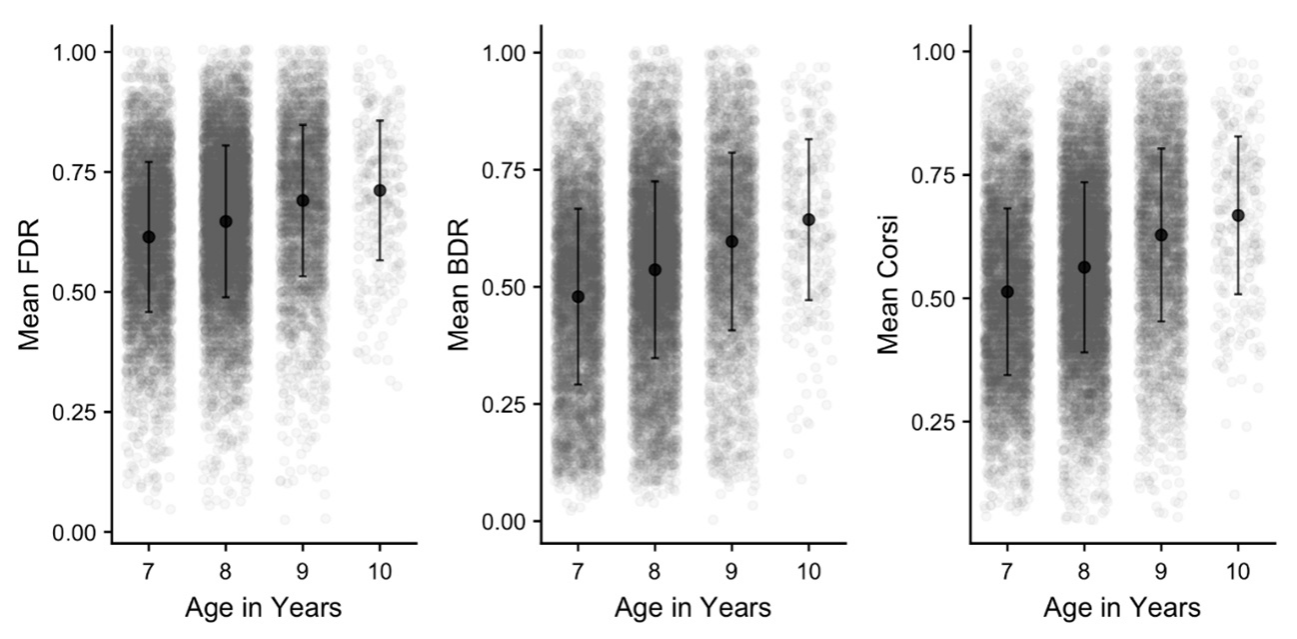
Mean proportion correct (and SD) in each working memory task and age group. Grey points denote individual children.

***Working memory.*** Performance on each of the working memory tasks was scored as the mean proportion of correct responses across all trials in the task. This is presented in Figure 13 for each task and age group. Age differences are formally analysed as part of the subsequent section examining sequence length, but it is clear that performance improves with age in each task. There is also substantial variation in performance within each age group, as indicated by the large SDs and spread of individual data points. Finally, the tasks themselves differ in performance levels, with FDR generally superior to Corsi. As befitting its status as a complex WM task, recall accuracy was lowest in BDR, even though this measure used a shorter range of sequence lengths.

**Figure 14.**
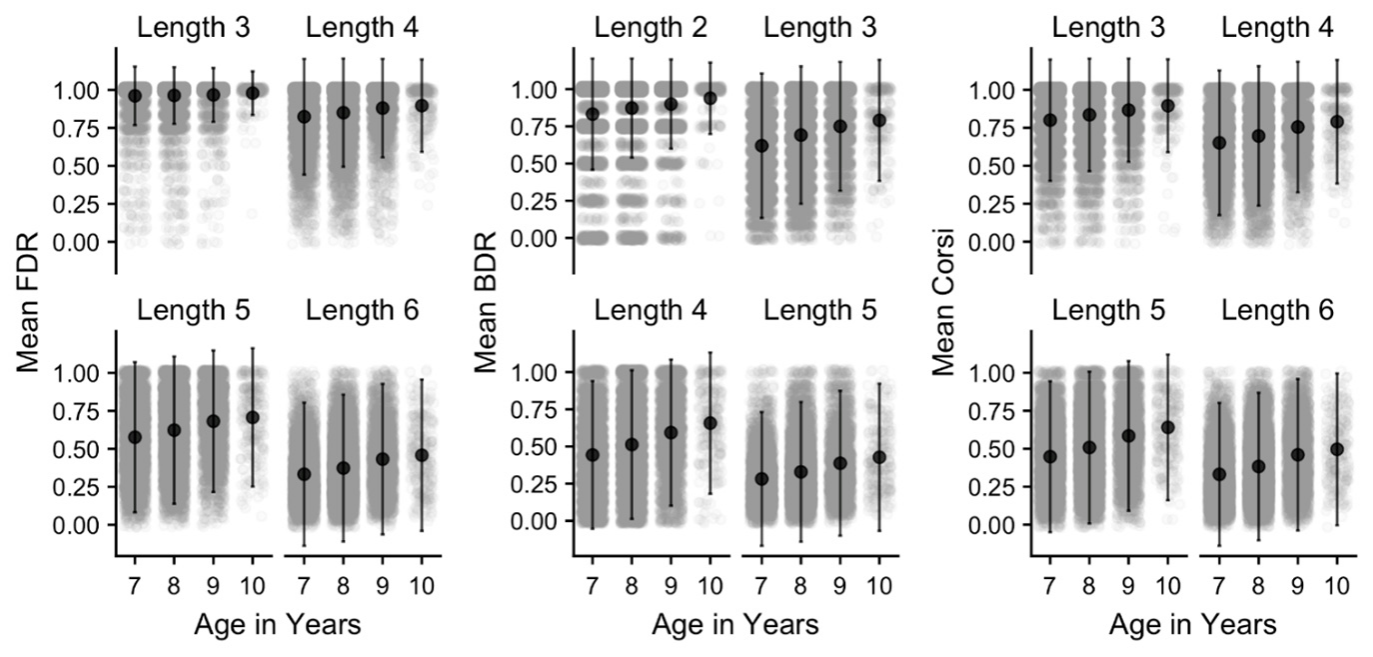
Mean proportion correct (and SD) in each working memory task and age group, separated by sequence length. Grey points denote individual children.

*Sequence length:* Performance was then examined as a function of sequence length, with mean performance for each task and age group illustrated in Figure 14. A set of 4x4 mixed ANOVA were carried out on each cognitive task, with sequence length as the within-subjects factor and age group as the between-subjects factor. For all three tasks, this revealed significant effects (at *p* < .001) of age group ( $\eta_{p}^{2}$ = FDR .03, BDR .05, Corsi .05), sequence length ( $\eta_{p}^{2}$ = FDR .44, BDR .28, Corsi .27) and the age group x sequence length interaction ( $\eta_{p}^{2}$ = .01 for all tasks). Thus, recall performance improved with age, and declined with sequence length, for all measures. Further comparisons (Bonferroni-corrected) revealed that all age groups differed from each other at all lengths, with the exception of the FDR task, where there were no significant age differences at length 3 after correction, or between age 9 and 10 at certain sequence lengths in each task ( *p* >.05). In general, age group effects were somewhat attenuated at the shortest sequence length for each task, indicating the higher performance levels at the easiest level of each task, and manifesting in the age group x sequence length interactions that were observed.

***Inhibition (Flanker).*** The inhibition task data were first trimmed by removing any reaction time that fell >3 SD above the mean across all participants and conditions. We also removed any child who achieved less than .25 correct across all trials. This resulted in the exclusion of 110 children from the sample, leaving N = 14,425.

**Figure 15.**
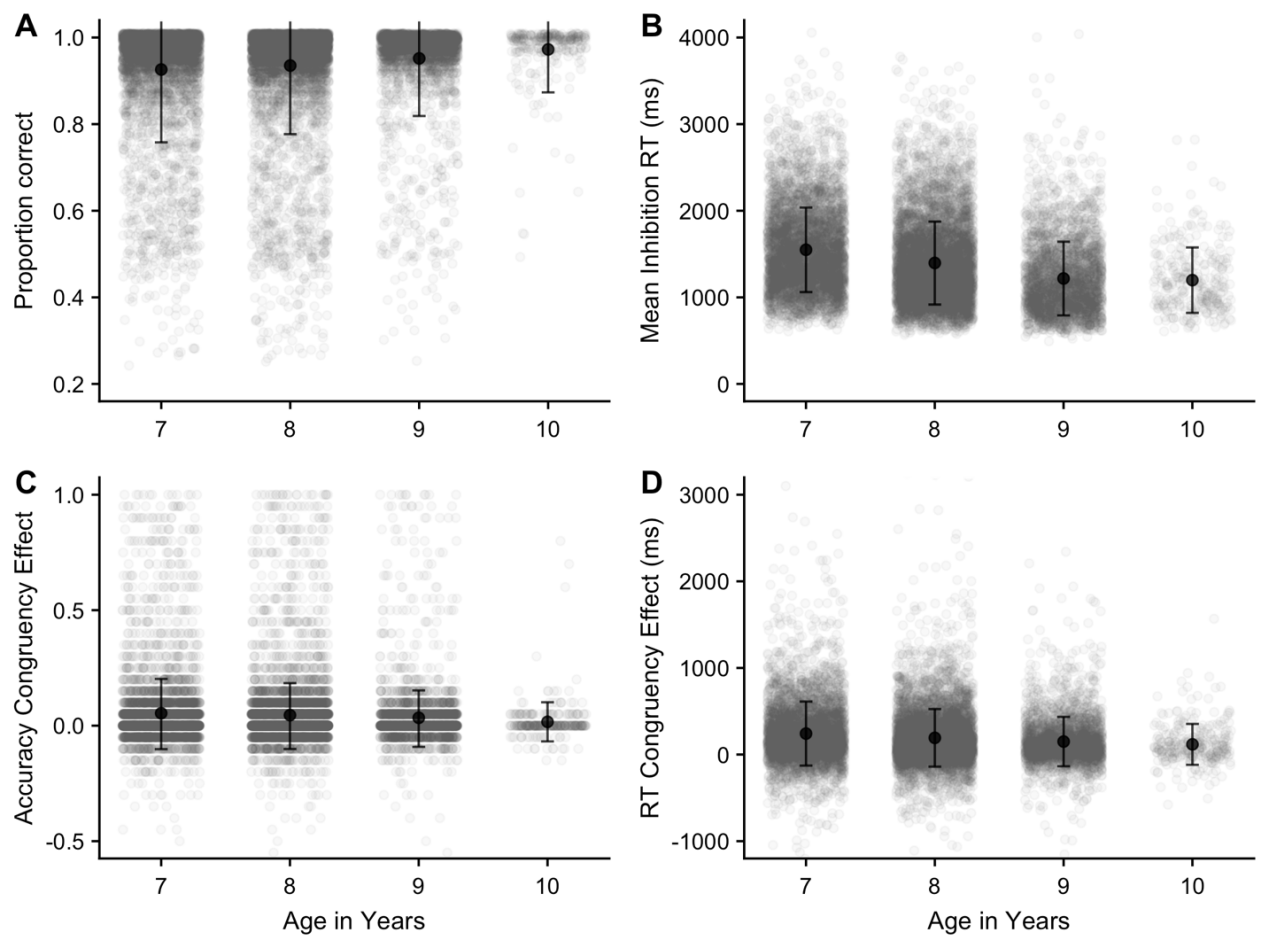
Mean proportion correct (A) and reaction time (B) in the Inhibition task (with SD). (C) (accuracy) and (D) (reaction time) display the mean difference between congruent and incongruent trial types.

Mean proportion correct and reaction time for correct responses are illustrated in Figure 15a and 15b, respectively. As expected, accuracy was typically very high on this task. One-way ANOVAs on each of these outcomes indicated that accuracy and response speed improved with age *(p* < .001) *,* with further comparisons showing significant differences ( *p* < .05) between all age groups (apart from age 9 and 10 in RT).

The primary outcome of interest on this task is the difference between RTs on congruent and incongruent trials (RT congruency effect), although we also investigated differences in accuracy between congruent and incongruent trials (accuracy congruency effect). Overall, responses were more accurate (proportion correct difference = .05, SE = .001) and faster (mean RT difference = 199ms, SE = 2.81) for congruent trials. This is plotted, by age group, in Figure 15, for accuracy (c) and reaction time (d). One-way ANOVAs showed that that the congruency effect on each of these outcome measures decreased with age ( *p* < .001); further comparisons showed significant differences ( *p* < .05) for both measures between all age groups apart from age 9 and 10.

**Figure 16.**
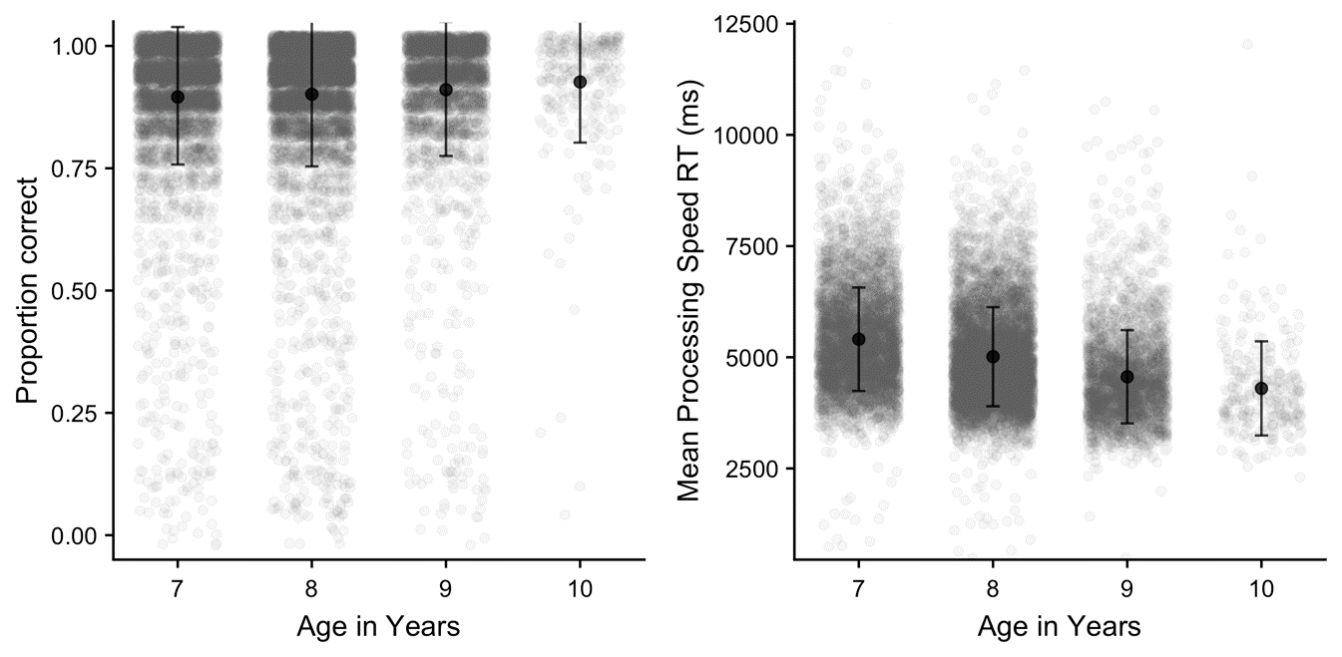
Mean proportion correct and reaction time in the processed speed task (with SD).

***Processing speed.*** The processing speed task data was trimmed by removing any reaction time that fell >3 SD above the mean across all participants. Mean proportion correct, and reaction time for correct responses are illustrated in Figure 16. The primary outcome variable of interest on this measure is reaction time to correct trials, although we also measured accuracy which was, as expected, typically very high. A one-way ANOVA on each outcome variable indicated significant age group effects in each case *(p* < .001) *;* further comparisons indicated all age groups significantly differed from each other *(p* < .05) *,* with only two exceptions (on proportion correct, 7–8 years, and 9–10 years).

**Figure 17.**
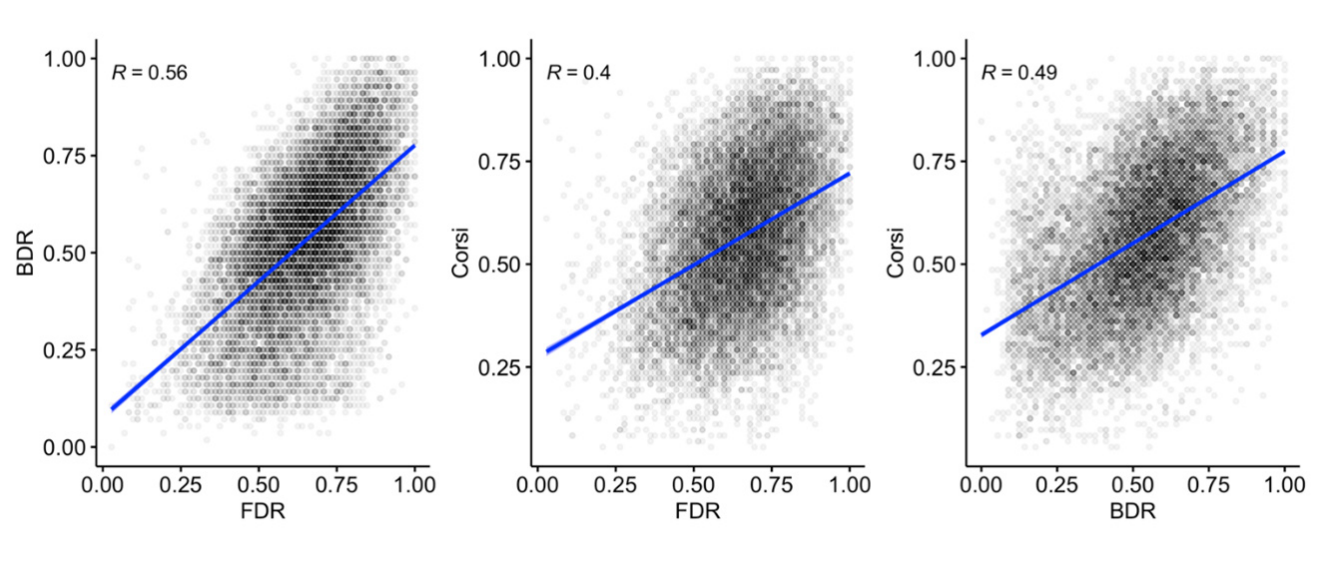
Scatterplots and correlations (Pearson’s R) illustrating the relationship between the three working memory measures (N=14,962).

***Relationships across cognitive tasks.*** Correlational analysis was carried out to examine the relationship between performance on different tasks. These were not corrected for age, though partial correlations controlling for age showed the same pattern of outcomes. Figure 17 shows the interrelationships between FDR, BDR, and Corsi. Positive correlations (significant at *p* < .001) are apparent between all measures.

**Figure 18.**
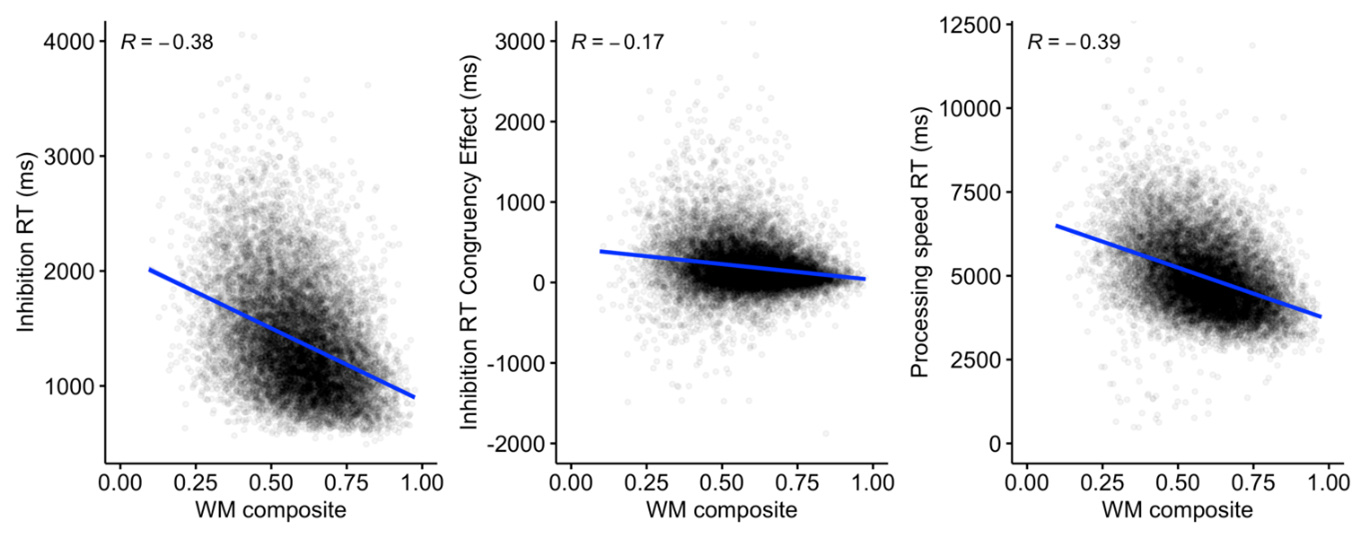
Scatterplots and correlations (Pearson’s R) illustrating the relationship between the working memory composite score, and reaction time outcomes for the inhibition and processing speed tasks (N=13,522).

A composite working memory score was then developed to examine the relationship with the reaction time outcomes from the inhibition and processing speed tasks. Figure 18 indicates a significant negative relationship *(p* < .001 for all outcomes) between working memory and inhibition RT, the inhibition congruent-incongruent RT difference, and processing speed RT.

## Discussion

In collecting data on the sensorimotor and cognitive performance from over 6,200 children participating in the Born in Bradford cohort, and a further 9,500 of their classmates, the Growing Up in Bradford project has succeeded in obtaining objective measures of these important aspects of cognitive and motoric functioning at a critical stage in children’s development. This initial paper established the methodological details of this data collection and illustrated that performance on all the assessments showed the changes one would expect in response to age and task difficulty. The predicted associations between tests of related sensorimotor functions, or cognitive abilities, were also observed. Furthermore, while these cross-sectional comparisons fit with the established literature on age-relevant cognitive and sensorimotor development, they also demonstrated heterogeneity within age groups. Indeed, it was apparent that within age-group variation was much larger than between age-group variation, particularly for certain tasks (e.g., working memory). This highlights the need to consider the profile of individual children in studying cognitive and sensorimotor development. It also demonstrates the challenges faced by teachers who may have children within a class that have, for example, a WM ability equivalent to a child several years younger through to several years older than the average age of the class.

### Sensorimotor tasks

The present analyses report findings that are consistent with our initial hypotheses and previous work conducted by Flatters *et al.* (2014), who also found that with increasing age performance across all three tasks *increased,* whilst increasing task difficulty *decreased* performance across all three tasks.

Specifically, previous work has demonstrated a significant three-way interaction between age, speed and condition for tracking ( Flatters *et al.,* 2014). Similar to our own findings, they also found that the benefit of slower target speed was greater with increasing age. When the target was moving at the fastest speed, age-related differences were still found but to a lesser extent than at the slower target speeds. This concurs with the view that increasing target speed is believed to influence shift away from feed *back* to feed *forward* control mechanisms for guiding action, which places increased reliance on predicting future target trajectory rather than using available online visual feedback; a more complex skill ( Ao *et al.,* 2015; van Roon *et al.,* 2008; Wolpert & Kawato, 1998). In this context, the benefits to be gained from providing additional visual feedback in the guide condition might be somewhat undermined by the need to process this additional information in a timely manner, leading to more reliance on feedforward responses.

Findings from the Aiming task were generally consistent with Flatters *et al.* (2014), who also found reduced age-related effects within the Jump trials. However, they did not find a significant interaction. Speculating on why such an interaction may have arisen in this dataset, it is instructive to note that whilst children possess the ability to produce on-line corrective movements from around eight years this is not believed to be fully automated until late childhood ( Mackrous & Proteau, 2016; Wilson & Hyde, 2013). Therefore, our data is likely reflective of both typical corrective aiming movement showing improvement across this specific age range, but at a slower rate for, more challenging, corrective movements. It would certainly be of interest in future research to see if these rates of improvement reversed, with further increases in typical aiming movement plateauing, whilst the capacity to make corrective movement continued to mature later into adolescence.

The Steering task (previously referred to as “Tracing” in Flatters *et al.,* 2014), showed a significant main effect of condition which had not been found in previous study. However, a potential explanation for this difference is the slight adaptation of the task for use in the Primary School Years data collection. In previous use, six trials were included within this task, alternating between Path A and Path B. However, the current version of the task was truncated to include only one trial for each task to reduce administration time, which could explain this discrepancy. Path A performance was significantly greater than Path B however the effect size was minimal which should also be considered. Additionally, Path B is identical to Path A in its proportions, with the only difference being that the path is flipped along its horizontal plane. Therefore, in Path B but not A it is partially occluded from view by the participants’ own hand for right-handed participants. The lack of this additional visual information could explain significantly poorer performance in this condition, albeit a difference of negligible magnitude. Further support of this hypothesis comes from findings from the Tracking task, where task performance is compromised when the visual guide is not provided.

Lastly, it was found that performance across all three CKAT tasks were significantly related. These relationships were found to be largest between the Tracking and Steering tasks. Whilst each CKAT task measures a distinct sensorimotor skill, this corroborates previous research that has found a reasonable degree of correlation between sub-tests on other standardised assessments of sensorimotor skill, such as the MABC-2 and the DCD-Q'07 ( Ellinoudis *et al.,* 2011; Parmar *et al.,* 2014).

### Cognitive tasks

Across all measures, the cognitive battery produced outcomes that were in line with the starting hypotheses. Firstly, age differences were apparent throughout, with children improving with age on accuracy and (where relevant) response speed on all tasks. These results were in line with established developmental changes in working memory and executive function ( Diamond, 2013; Gathercole, 1999). The data also revealed that within-group variation was much larger than between-group variation, particularly for the working memory tasks. This suggests that individual differences need to be considered within the context of age-related improvements.

Sequence length effects were observed within each working memory task, and these were consistent across the different age groups, indicating that these measures were effective in capturing the limited capacity of working memory. Recall accuracy was also somewhat lower in BDR, relative to FDR or Corsi (even though BDR used a shorter set of lengths), reflecting the more difficult and processing-intensive nature of this task. Finally, for the inhibition (Flanker) task, significant congruency effects were observed in both reaction time and accuracy, indicating that the task was effective in indexing the greater executive cost of responding to a visual stimulus in the presence of incongruent flanker items. Furthermore, this cost decreased from the younger to older children, in line with age-related development in executive function.

The predicted positive interrelationships were observed between the three working memory measures, which remained after controlling for age, with some indication of a closer positive relationship between FDR and BDR. These patterns are likely to reflect a combination of shared and separable components operating within and across domains in working memory. Working memory performance was also related to inhibition and processing speed, including a moderate but still highly significant correlation between the working memory composite score and the magnitude of the inhibition congruency effect.

### Applications, strengths and limitations

In the context of the Growing up in Bradford study, these measures are particularly valuable because, for BiB participants, they can be linked with contemporaneous data that has also been collected regarding their social and emotional wellbeing, growth, adiposity, and cardiometabolic health ( Bird *et al.,* 2019). Longitudinally, there is also the capacity to link these cross-sectional data with data at earlier time points from BiB participants. This includes biological samples and maternal and paternal questionnaire responses collected at baseline ( Wright *et al.*, 2013), routine health ( Bishop *et al.,* 2018) and educational data ( Pettinger *et al.,* 2020) that the cohort has permission to access, and further data collected from sub-samples of the cohort as part of nested research projects (e.g. the BiB-1000 sub-cohort, see Bryant *et al.,* 2013). Repeated measurement of cognition and sensorimotor ability in adolescence is also being planned as part of the next phase of data collection within the cohort. Further, the sensorimotor data reported here can be linked with the sensorimotor data collected when the children were 4–5 years old, within the Starting Schools project (see Shire *et al.,* 2020). Altogether this represents a rich set of data on children’s health, wellbeing and development that is an invaluable resource for future research. It will allow closer consideration of how participating children’s sensorimotor control and cognitive abilities integrate, influence, and are influenced by other factors in the course of their development.

The scale and objectivity of these assessments represent are a strength of this work. In particular, the use of precise, computerised assessments methods to measure children’s sensorimotor control at different time points are unique to the BiB cohort. Whilst other birth cohorts have attempted to assess their participant’s motor function, this is typically only captured via more subjective parental reports, collected at a much earlier age, reporting on when children accomplish specific gross motor milestones, such as in the Millenium Cohort Study (see Kelly *et al.,* 2006). The Avon Longitudinal Study of Parents and Children (ALSPAC) is the only other birth cohort study we are aware of to attempt a brief standardised assessment of motor function, even then only doing so at single time point in development, 7–8 years of age ( Lingam *et al.,* 2010).

In demonstrating the capacity to undertake relatively brief, laboratory quality, objective measures of cognition and sensorimotor control within a community setting (i.e. over eighty schools across four years), and feedback the results of these assessments to teachers in a timely and informative fashion, this project also demonstrates the potential applied value of using such assessments at scale within health and education services. For example, the data collected here on cognitive and sensorimotor functioning will be important for identifying children who show deficits in these core developmental constructs, and who will therefore be at risk for educational under-achievement and poorer longer-term outcomes. Fine motor skills can be improved by training ( Sugden *et al;*, 2013), and poor cognitive ability can be mitigated by appropriate classroom support ( Gathercole & Alloway, 2008). Thus, these data, and the assessment tools developed for use within this cohort to collect them, can help to inform subsequent interventions that aim to improve the long-term physical and mental health outcomes for children.

However, it should also be acknowledged that neither the specific cognitive nor sensorimotor assessments used here constitute a comprehensive of either of these broad constructs. Collecting large-scale data via school-based assessment meant we were operating under tight time constraints. As such, we selected tasks that represent fundamental sensorimotor and cognitive constructs with a strong body of evidence linking them to key outcomes. Additional nested projects are planned within the BiB cohort to supplement the data presented here. Further, via Connected Bradford, we can link data to school records, including measures of language, literacy and physical literacy (e.g., phonics, EAL status, reading attainment, gross motor skills).

In summary, the collection of objective computerised measures of a range of cognitive and sensorimotor functions at 7–10 years of age in over 15,500 children has created a comprehensive dataset that can be used to answer more specific questions about the development of these constructs, and can facilitate future studies of the relationship between cognition and motor function. These data also have real world applications, such as identifying children within the education system in need of additional support, and providing teachers with feedback on individual children to enable schools to make informed decisions about how to best tailor their approach to supporting individual learners. In particular, in collecting these measures on over 6,200 participants in the Born in Bradford study, the project also greatly enriches the longitudinal dataset available on the participants involved in this birth cohort study. This will help build a deeper understanding of the complex relationships between cognition, sensorimotor ability and other key aspects of childhood development. 

## Data availability

The data referenced by this article are under copyright with the following copyright statement: Copyright: ï¿½ 2021 Hill LJ et al.

Data associated with the article are available under the terms of the Creative Commons Zero "No rights reserved" data waiver (CC0 1.0 Public domain dedication). 

### Underlying data

Scientists are encouraged to make use of the BiB data, which are available through a system of managed open access.

Before you contact BiB, please make sure you have read our Guidance for Collaborators. Our BiB executive review proposals on a monthly basis and we will endeavour to respond to your request as soon as possible. You can find out about all of the different datasets which are available here. If you are unsure if we have the data that you need please contact a member of the BiB team ( borninbradford@bthft.nhs.uk).

Once you have formulated your request please complete the ‘Expression of Interest’ form available here and email the BiB research team ( borninbradford@bthft.nhs.uk).

If your request is approved, we will ask you to sign a collaboration agreement; if your request involves biological samples, we will ask you to complete a material transfer agreement.

### Extended data

Open Science Framework: Markdown report containing explanation of how to derive the sensorimotor variables from the raw CKAT output. https://doi.org/10.17605/OSF.IO/TSVX6 ( Shire, 2020).

Extended data are available under the terms of the Creative Commons Zero "No rights reserved" data waiver (CC0 1.0 Public domain dedication).

## Notes

^1^The UPN is a 13-character code allocated on entry into formal education (typically primary school) that identifies a child at a national level within education databases

^2^At the end of each session conducted in a school the observing researcher had the option to add a free text Field Note to the data collected, to be saved alongside it. Normally this was used to note any unusual circumstances that had arisen during that session. 
