# Supplementary material for: Large-scale assessment of 7-11-year-olds’ cognitive and sensorimotor function within the Born in Bradford longitudinal birth cohort study
Source: Wellcome Open Res. 2022 Feb 9;6:53. Originally published 2021 Mar 10. [Version 2] doi: 10.12688/wellcomeopenres.16429.2 (PMC10511857; doi:10.12688/wellcomeopenres.16429.2)
Supplement: Peer review history

# Reviewer report: Reviewer response for version 2

**DOI:** 10.21956/wellcomeopenres.19546.r58758

**Published:** 2023-09-20

**Authors:** Sinead Rhodes

**Affiliations:** 1 The University of Edinburgh, Edinburgh, Scotland, UK

This study reports on an important area of focus namely cognitive development during childhood. The findings build on previous reports from other studies that highlight the issue of within age variability in cognitive development especially working memory. 

 The large sample size and opt-out recruitment approach is a clear strength of the study. 

 It would be good to hear of further information about the recruitment in relation to SEND characteristics. It is estimated that 15-20% of children are neurodivergent. Did the researchers include any assessment of this? Is their sample a mixed typical and SEND sample? It seems that some neurodivergent children may be excluded due to teacher report but it is not clear as to how systematic this was. 

 The interpretation of findings is very clear and has important implications for understanding and supporting children with age-inappropriate cognitive development.

Is the work clearly and accurately presented and does it cite the current literature?

Yes

If applicable, is the statistical analysis and its interpretation appropriate?

Yes

Are all the source data underlying the results available to ensure full reproducibility?

Yes

Is the study design appropriate and is the work technically sound?

Yes

Are the conclusions drawn adequately supported by the results?

Yes

Are sufficient details of methods and analysis provided to allow replication by others?

Yes

Reviewer Expertise:

Cognitive development

I confirm that I have read this submission and believe that I have an appropriate level of expertise to confirm that it is of an acceptable scientific standard.

# Reviewer report: Reviewer response for version 2

**DOI:** 10.21956/wellcomeopenres.19546.r57687

**Published:** 2023-09-20

**Authors:** Mariza Kampouri

**Affiliations:** 1 Karolinska Institutet, Solna, Sweden

The aim of the study was to report on results of neuropsychological assessment (cognitive and sensorimotor function) at 7–11-year-olds in the BiB longitudinal birth cohort study.

 This article comprehensively presents data on the sensory-motor and cognitive skills of a large cohort of children as part of an ongoing longitudinal study. The study involved over 15,000 children who underwent testing for sensory-motor skills, specifically tracking, aiming, and steering, as well as cognitive functions, including working memory, inhibition, and processing speed, through various tasks. The results indicate an age-related increase in all the assessed skills and support positive correlations between sensory-motor and cognitive measures. These findings are in line with established theories and consensus on neuropsychological development. 

 The article is well-written and engaging, providing valuable insights into neuropsychological development within a large population-based cohort study. Although it doesn't address a specific research gap, it serves as a detailed and informative reference for readers seeking to understand the neurodevelopmental data of the cohort. 

 The manuscript is comprehensive and detailed on the procedures that were followed. The analyses are straightforward and presented clearly. I have no further comments.

Is the work clearly and accurately presented and does it cite the current literature?

Yes

If applicable, is the statistical analysis and its interpretation appropriate?

Yes

Are all the source data underlying the results available to ensure full reproducibility?

Yes

Is the study design appropriate and is the work technically sound?

Yes

Are the conclusions drawn adequately supported by the results?

Yes

Are sufficient details of methods and analysis provided to allow replication by others?

Yes

Reviewer Expertise:

Epidemiology; Psychology; Child health and development

I confirm that I have read this submission and believe that I have an appropriate level of expertise to confirm that it is of an acceptable scientific standard.

# Reviewer report: Reviewer response for version 1

**DOI:** 10.21956/wellcomeopenres.18078.r43008

**Published:** 2021-04-22

**Authors:** James Tresilian

**Affiliations:** 1 Department of Psychology, University of Warwick, Coventry, UK

The manuscript reports on the methods and results of a cross-sectional study of sensorimotor and cognitive performance in over 15,000 primary school age children – a significant feat of data collection. The data are reported by age in years. The vast majority of the children were aged 7, 8 or 9 years, there were a relatively small number of 10 year olds (Table 1). All children were tested on the same battery of sensorimotor and cognitive tests and so the study provides a substantial data set concerning childrens’ performance on these tests across the age range tested.

 The sensorimotor data are a particularly important component of the data set. The computerized test battery that was used involved the collection of movement end-point position data at a suitably high sampling rate (I assume that these data will be available from the repository), allowing subsequent computation of movement speed, acceleration and other kinematic variables. This permits the fine grained analysis of the actual movement performance itself, rather than simply the outcome of the movement. The possibility of such fine grained analysis in such a large group is something few other studies can offer and so these data represent a significant resource in and of themselves. The additional cognitive performance data available for the whole group and the genetic, social and geographical data for the Born in Bradford cohort group, makes this a truly unique resource with great potential, especially if followed through into adolescence as indicated in the manuscript.

 The manuscript provides details concerning the data acquisition/testing procedures, the groups of children studied and the recruitment process together with a preliminary analysis of some main features of the data. As such it provides a useful and important starting point for future secondary data analysis work. Obviously, one could ask why some analyses were conducted and others not or why some analyses were more detailed and others less so. Given the aims of the study and the preliminary or indicative nature of the analysis, I think the choice of analyses is a reasonable one and this is not the time/place to quibble about perceived omissions. The authors appropriately comment on this in the discussion section (pages 18-19), though this comment would arguably be better placed in the introduction.

 I have no doubt that this is a valuable report, I have a few specific and relatively minor comments that might help the authors improve the clarity of their exposition. I list these in order of occurrence below.

1.  At the bottom of page 3 the authors make the claim that the sensorimotor tasks used are “representative of fundamental coordinative abilities”, these being tracking, aiming and steering. I was not sure about the validity of this claim. It seems that the claim amounts to saying that tracking, aiming and steering are fundamental coordinative abilities. On the face of it, this seems plausible mainly because aiming underlies many different kinds of activities from reaching to pick something up to playing sports and steering is fundamental to getting around in the world. However, the kind of steering assessed in the test task is not that kind of steering – the task was actually a kind of computer version of the pass a loop along a twisted piece of wire game, where a bell rings if you touch the wire with the loop. It doesn’t have a lot to do with steering your way around the environment: indeed, the task was called ‘tracing’ in an earlier study, which is arguably a more accurate description of what it is.  In addition, use of the term ‘coordination’ is unclear given that coordination per se doesn’t seem to have been measured/assessed. I wouldn’t want to argue that the skills underlying performance in these tasks aren’t important ones – I’m sure they are – it’s just that the task names ‘tracking’ and ‘steering’ don’t accurately describe what went on. The tracking task involved following the path of a moving target and the steering task involved tracing a path. I think it would be helpful to the reader to make this clear.
2. In the same paragraph (p. 3) that aiming movements rely on “fast implementation of online corrections” in the production of “ballistic” responses. The term ‘ballistic’ refers to movements produced without any on-line corrections, so some kind of adjustment to the text is warranted here. The final sentence of the paragraph is also rather confusing, I wasn’t sure what the authors were saying here and what the ‘timing constraints’ are. Some revision of this paragraph is, I think, needed.
3. On page 4 there is a short paragraph describing the ‘inhibitory control’ task. It is true that inhibitory control refers to situations of response suppression (including impulse suppression) and to the inhibition of irrelevant stimuli. However, the term is more commonly used for the first of these and the term ‘selective attention’ is used for the latter. The task used (flanker task) involves inhibition of irrelevant stimuli and so is typically described as a selective attention task. It might help the reader to make this explicit.
4. In the introduction, the BiB cohort is stated to comprise 13,776 children. Only about 6200 of these participated in the testing reported in the manuscript. It is not clear why – perhaps some of the children were too old or too young? Perhaps some weren’t in the schools recruited? A bit of information about why less than half the BiB cohort participated would be useful, especially for a reader like me who doesn’t know much about the cohort. On a related matter, it is interested to note from Table 2 that in all school years the proportion of recruited BiB children who were assessed is lower than the proportion of recruited non-BiB children who were assessed (about 2% lower on average). On the face of it, this seems odd, given that the BiB children had been previously recruited into the cohort and were presumably expecting to be tested. Is there any known reason for the discrepancy?
5. On page 5 where the design and measurements are described, it is stated that the procedure was “very similar” to that previously used for the ‘Starting Schools’ study. It seems that this means only that more time was allowed for the cognitive tasks in the present study. Is this the case? If it is, then the procedure was identical (rather than very similar) for the sensorimotor tasks. I think it would be clearer if it were stated that procedures were identical except for the added time. Was there any reason for the added time?
6. On page 10 it is stated that task data were analyzed with ANOVAs with “follow-up comparisons”. Were these post hoc comparisons? If they were, were they only conducted when the relevant main effects or interactions were significant? Given that these comparisons were often between different age groups, it would be reasonable to assume that they could have been pre-planned, in which case they are arguably not ‘follow-up’ comparisons at all. In fact, the main effects of age group that would be predicted are actually trends for improvement over years. This would imply that ANOVA trend analysis and pre-planned comparisons would be the appropriate statistical approach.
7. It is stated on the top of page 16 that a “composite working memory score” was developed. I may have missed something, but I couldn’t see how the score was developed or what exactly it involved.
8. Early in the discussion section (page 17), the authors comment on the large within age group variability. The data presentation used in the graphs nicely shows this variability, which stands out as a significant feature of the data. What isn’t obvious is the extent to which a significant proportion of the variability is simply a result of the variability in age within each group. Dividing into groups based on age in years is a coarse grained means of division – a 7 year old child could be any age from exactly 7 years old to 7 years 11 months and 29/30 days old. Thus the difference in age between 7 two 7 year olds could be nearly a year, whereas the difference in age between a 7 year old and an 8 year old could be as little as one day. This large variability in age (as a proportion of the entire age range) could well be the main contributory factor to the within group variability in performance. A finer grained analysis of age-groups would, of course, be possible given that the exact age data are available. The authors should comment further on these matters.

Is the work clearly and accurately presented and does it cite the current literature?

Yes

If applicable, is the statistical analysis and its interpretation appropriate?

Partly

Are all the source data underlying the results available to ensure full reproducibility?

Yes

Is the study design appropriate and is the work technically sound?

Yes

Are the conclusions drawn adequately supported by the results?

Yes

Are sufficient details of methods and analysis provided to allow replication by others?

Yes

Reviewer Expertise:

Human sensorimotor control

I confirm that I have read this submission and believe that I have an appropriate level of expertise to confirm that it is of an acceptable scientific standard.

# Response

**Published:** 2022-01-26

**Authors:** Katy Shire

**Affiliations:** Bradford Hospitals National Health Service Trust, UK

*Comment:*

 At the bottom of page 3 the authors make the claim that the sensorimotor tasks used are “representative of fundamental coordinative abilities”, these being tracking, aiming and steering. I was not sure about the validity of this claim. It seems that the claim amounts to saying that tracking, aiming and steering are fundamental coordinative abilities. On the face of it, this seems plausible mainly because aiming underlies many different kinds of activities from reaching to pick something up to playing sports and steering is fundamental to getting around in the world. However, the kind of steering assessed in the test task is not that kind of steering – the task was actually a kind of computer version of the pass a loop along a twisted piece of wire game, where a bell rings if you touch the wire with the loop. It doesn’t have a lot to do with steering your way around the environment: indeed, the task was called ‘tracing’ in an earlier study, which is arguably a more accurate description of what it is. In addition, use of the term ‘coordination’ is unclear given that coordination per se doesn’t seem to have been measured/assessed. I wouldn’t want to argue that the skills underlying performance in these tasks aren’t important ones – I’m sure they are – it’s just that the task names ‘tracking’ and ‘steering’ don’t accurately describe what went on. The tracking task involved following the path of a moving target and the steering task involved tracing a path. I think it would be helpful to the reader to make this clear. In the same paragraph (p. 3) that aiming movements rely on “fast implementation of online corrections” in the production of “ballistic” responses. The term ‘ballistic’ refers to movements produced without any on-line corrections, so some kind of adjustment to the text is warranted here. The final sentence of the paragraph is also rather confusing, I wasn’t sure what the authors were saying here and what the ‘timing constraints’ are. Some revision of this paragraph is, I think, needed.

*Response:*

 We’ve reflected on the reviewers comments here and extensively redrafted this paragraph, which is meant to serve as a brief justification of our selection of CKAT as the specific tool we chose to use as a measure sensorimotor function. We have removed the more speculative claims they commented on. Instead, we now justify our selection of CKAT based on prior precedents, and other relevant findings from the research literature. 

 Later, in the Methods, we’ve also clarified that in earlier versions of CKAT the ‘Steering’ subtask was known as ‘Tracing’. It was first renamed Steering in Giles et al. (2018) and has since been referred to by this name in all subsequent publications. One of the co-creators of CKAT explained to us that they proposed this renaming after observing that performance on this task was predictive of participants steering performance in a driving simulator (Raw et al., 2012).

 Here, we have simply chosen to refer to these tasks by the names given to them by their creators and that are in current usage (i.e. Tracking, Aiming and Steering). 

*Comment:*

 On page 4 there is a short paragraph describing the ‘inhibitory control’ task. It is true that inhibitory control refers to situations of response suppression (including impulse suppression) and to the inhibition of irrelevant stimuli. However, the term is more commonly used for the first of these and the term ‘selective attention’ is used for the latter. The task used (flanker task) involves inhibition of irrelevant stimuli and so is typically described as a selective attention task. It might help the reader to make this explicit.

*Response:*

 We thank the reviewer for pointing out the inconsistencies with labelling that exist for these types of task. We have added some text to the paper to acknowledge this.

*Comment:*

 In the introduction, the BiB cohort is stated to comprise 13,776 children. Only about 6200 of these participated in the testing reported in the manuscript. It is not clear why – perhaps some of the children were too old or too young? Perhaps some weren’t in the schools recruited? A bit of information about why less than half the BiB cohort participated would be useful, especially for a reader like me who doesn’t know much about the cohort. On a related matter, it is interested to note from Table 2 that in all school years the proportion of recruited BiB children who were assessed is lower than the proportion of recruited non-BiB children who were assessed (about 2% lower on average). On the face of it, this seems odd, given that the BiB children had been previously recruited into the cohort and were presumably expecting to be tested. Is there any known reason for the discrepancy?

*Response:*

 We recruited on a school-by-school basis, targeting those schools with the highest proportion of BiB children initially. This necessarily meant we could not aim to recruit all BiB children.

 There is no known reason for this difference. Table 2 relates only to children who had been ‘recruited’ i.e. not withdrawn by their parents. BiB and non-BiB children were subject to exactly the same procedure and given the same information in school.

*Comment:*

 On page 5 where the design and measurements are described, it is stated that the procedure was “very similar” to that previously used for the ‘Starting Schools’ study. It seems that this means only that more time was allowed for the cognitive tasks in the present study. Is this the case? If it is, then the procedure was identical (rather than very similar) for the sensorimotor tasks. I think it would be clearer if it were stated that procedures were identical except for the added time. Was there any reason for the added time?

 This has been adjusted in the manuscript. The procedure was identical for the sensorimotor tasks, the additional time was only for the cognitive tasks (which were not done as part of the Starting School study)

 On page 10 it is stated that task data were analyzed with ANOVAs with “follow-up comparisons”. Were these post hoc comparisons? If they were, were they only conducted when the relevant main effects or interactions were significant? Given that these comparisons were often between different age groups, it would be reasonable to assume that they could have been pre-planned, in which case they are arguably not ‘follow-up’ comparisons at all. In fact, the main effects of age group that would be predicted are actually trends for improvement over years. This would imply that ANOVA trend analysis and pre-planned comparisons would be the appropriate statistical approach.

*Response:*

 This has now been adjusted in the manuscript, to indicate that the age group comparisons were planned rather than post hoc.

 Regarding the final point, we would acknowledge that other forms of analytic approach are available, but would reiterate that this manuscript aimed to provide a first description of methods and basic outcomes.

*Comment:*

 It is stated on the top of page 16 that a “composite working memory score” was developed. I may have missed something, but I couldn’t see how the score was developed or what exactly it involved.

*Response:*

 This detail has now been added.

*Comment:*

 Early in the discussion section (page 17), the authors comment on the large within age group variability. The data presentation used in the graphs nicely shows this variability, which stands out as a significant feature of the data. What isn’t obvious is the extent to which a significant proportion of the variability is simply a result of the variability in age within each group. Dividing into groups based on age in years is a coarse grained means of division – a 7 year old child could be any age from exactly 7 years old to 7 years 11 months and 29/30 days old. Thus the difference in age between 7 two 7 year olds could be nearly a year, whereas the difference in age between a 7 year old and an 8 year old could be as little as one day. This large variability in age (as a proportion of the entire age range) could well be the main contributory factor to the within group variability in performance. A finer grained analysis of age-groups would, of course, be possible given that the exact age data are available. The authors should comment further on these matters.

*Response:*

 We completely agree with the reviewer’s comments. This type of detailed analysis will be included in subsequent papers, where we do more fine-grained analyses of all the data. The aim of the current manuscript is to present the details of the methodology, and to present some initial, high-level analysis of the data.

 When we look at age by months, we still see very high levels of individual variability within each of the groups (i.e., each separate month), similar to that when looking at it by age in years. We have added a comment in the manuscript signposting the reader to the fact that additional papers will be published looking at more detailed analyses.

# Reviewer report: Reviewer response for version 1

**DOI:** 10.21956/wellcomeopenres.18078.r43009

**Published:** 2021-04-22

**Authors:** Evie Vergauwe, Beatrice Valentini

**Affiliations:** 1 Department of Psychology and Educational Sciences, University of Geneva, Geneva, Switzerland

This article reports the observations regarding sensory-motor and cognitive skills of a large group of children that were tested as part of a large-scale longitudinal study. The current observations are based on >15’000 children who were tested at one time point using tasks to assess three sensory-motor skills (tracking, aiming and steering) and three cognitive capacities (working memory, inhibition and processing speed). Overall, the results show that there is an age-related increase in all of these skills, in line with most theories and studies that are currently available. Furthermore, as expected, all sensory-motor measures were positively correlated and all cognitive measures were positively correlated.

 Overall, this article is interesting and well written. The article is not really addressing a novel question, nor is it addressing debated issues in the field. Indeed, to our knowledge, not many researchers would have predicted a different outcome. However, there is value in this type of large-scale studies providing us with a huge amount of interesting data for the research community. Moreover, as the authors explain in the introduction, there are also other types of data available for a subsample of the current sample of primary school children (e.g., social and emotional wellbeing, growth, adiposity, and cardiometabolic health). In that regard, we find it unfortunate that the data do not seem to be as openly accessible as we expected. Firstly, the hyperlink in page 19 (i.e., “You can find out about all of the different datasets which are available here”) is a dead link. Secondly, it appears that, to have access to the data reported in the current article, one needs to go through to a procedure to be granted access, which involves the completion of an “expression of interest” form and then signing a collaboration agreement (which seems to come with specific, rather strict, rules about authorship and publication).

 Some further, more minor points that deserve attention are listed below (in no particular order) :

- The reported observations are in essence based on one time point of a longitudinal study and are thus the result of a cross-sectional portion of a longitudinal study. We feel that this should be more explicitly stated in the manuscript, as there are currently several points at which the reader may be confused. Moreover, given that the current report is not presenting any longitudinal data, we think the introduction and general discussion should mainly focus on that, rather than describing the benefits and potential impact of the longitudinal study that also includes other measures. The current report is not presenting longitudinal data, nor is it presenting any additional measure beyond sensory-motor and cognitive performance.
- It would be useful to know what software was used to program the different tasks.
- For the Aiming task : the text describes a task involving green circles, but Figure 2 shows red circles.
- Figure 8 is not entirely clear to us. Perhaps more explanation is needed here. For example, for Year 16/17, 95 schools seemed to have been approached, 2 declined, and this resulted in 51 schools being recruited. It is unclear what happened to the remaining 42 schools who did not decline.
- For the working memory tasks, the method section mentioned « the primary outcome variable was mean proportion correct and the secondary outcome variable was mean reaction time (RT). », but we could not find any information or analysis on the secondary variable, i.e., on RT.
- It is unclear to us why Age was analyzed as a categorical variable in this study.

Is the work clearly and accurately presented and does it cite the current literature?

Yes

If applicable, is the statistical analysis and its interpretation appropriate?

Yes

Are all the source data underlying the results available to ensure full reproducibility?

Partly

Is the study design appropriate and is the work technically sound?

Yes

Are the conclusions drawn adequately supported by the results?

Yes

Are sufficient details of methods and analysis provided to allow replication by others?

Yes

Reviewer Expertise:

Cognition, Working memory, cognitive development

We confirm that we have read this submission and believe that we have an appropriate level of expertise to confirm that it is of an acceptable scientific standard, however we have significant reservations, as outlined above.

# Response

**Published:** 2022-01-26

**Authors:** Katy Shire

**Affiliations:** Bradford Hospitals National Health Service Trust, UK

*Comment:*

 Overall, this article is interesting and well written. The article is not really addressing a novel question, nor is it addressing debated issues in the field. Indeed, to our knowledge, not many researchers would have predicted a different outcome. However, there is value in this type of large-scale studies providing us with a huge amount of interesting data for the research community. Moreover, as the authors explain in the introduction, there are also other types of data available for a subsample of the current sample of primary school children (e.g., social and emotional wellbeing, growth, adiposity, and cardiometabolic health). In that regard, we find it unfortunate that the data do not seem to be as openly accessible as we expected. Firstly, the hyperlink in page 19 (i.e., “You can find out about all of the different datasets which are available here”) is a dead link. Secondly, it appears that, to have access to the data reported in the current article, one needs to go through to a procedure to be granted access, which involves the completion of an “expression of interest” form and then signing a collaboration agreement (which seems to come with specific, rather strict, rules about authorship and publication).

*Response:*

 All data relating to the Born in Bradford cohort is subject to the same restrictions and the authors do not have the ability to alter these. The BiB executive welcome collaborators to apply to use all the data collected and can provide support in navigating the process.

 The hyperlink has been updated to link directly to the data dictionary page, rather than the top level page for the research data section on the BiB website.

*Comment:*

 The reported observations are in essence based on one time point of a longitudinal study and are thus the result of a cross-sectional portion of a longitudinal study. We feel that this should be more explicitly stated in the manuscript, as there are currently several points at which the reader may be confused. Moreover, given that the current report is not presenting any longitudinal data, we think the introduction and general discussion should mainly focus on that, rather than describing the benefits and potential impact of the longitudinal study that also includes other measures. The current report is not presenting longitudinal data, nor is it presenting any additional measure beyond sensory-motor and cognitive performance.

*Response:*

 We have updated the abstract and the introduction to help make this distinction more explicit.

*Comment:*

 It would be useful to know what software was used to program the different tasks.

*Response:*

 This detail has been added for both the motor and cognitive tasks in the manuscript.

*Comment:*

 For the Aiming task : the text describes a task involving green circles, but Figure 2 shows red circles.

*Response:*

 The dots would initially be presented as red, and turn green as they were successfully reached by the participant. We agree this was confusing in the manuscript, and the text has been updated to avoid this confusion.

*Comment:*

 Figure 8 is not entirely clear to us. Perhaps more explanation is needed here. For example, for Year 16/17, 95 schools seemed to have been approached, 2 declined, and this resulted in 51 schools being recruited. It is unclear what happened to the remaining 42 schools who did not decline.

*Response:*

 We agree this is unclear from the figure, and have added some in-text description to address this. Schools that were approached, but did not respond, were not counted as actively ‘declining’.

*Comment:*

 For the working memory tasks, the method section mentioned « the primary outcome variable was mean proportion correct and the secondary outcome variable was mean reaction time (RT). », but we could not find any information or analysis on the secondary variable, i.e., on RT.

*Response:*

 RT is not reported in the manuscript. We have now noted this in the method section.

*Comment:*

 It is unclear to us why Age was analyzed as a categorical variable in this study.

*Response:*

 The current paper’s aim is to present a detailed description of the methodology, and some initial, high-level analyses by key variables. More detailed analyses will be presented in future papers, including detailed analyses using age in months as a continuous variable. We have added some text on p.25 relating to this issue.
